# Ploidy-Regulated Variation in Biofilm-Related Phenotypes in Natural Isolates of *Saccharomyces cerevisiae*

**DOI:** 10.1534/g3.114.013250

**Published:** 2014-07-24

**Authors:** Elyse A. Hope, Maitreya J. Dunham

**Affiliations:** Department of Genome Sciences, University of Washington, Seattle, Washington 98195

**Keywords:** biofilm, *Saccharomyces cerevisiae*, natural isolates, flocculation

## Abstract

The ability of yeast to form biofilms contributes to better survival under stressful conditions. We see the impact of yeast biofilms and “flocs” (clumps) in human health and industry, where forming clumps enables yeast to act as a natural filter in brewing and forming biofilms enables yeast to remain virulent in cases of fungal infection. Despite the importance of biofilms in yeast natural isolates, the majority of our knowledge about yeast biofilm genetics comes from work with a few tractable laboratory strains. A new collection of sequenced natural isolates from the *Saccharomyces* Genome Resequencing Project enabled us to examine the breadth of biofilm-related phenotypes in geographically, ecologically, and genetically diverse strains of *Saccharomyces cerevisiae*. We present a panel of 31 haploid and 24 diploid strains for which we have characterized six biofilm-related phenotypes: complex colony morphology, complex mat formation, flocculation, agar invasion, polystyrene adhesion, and psuedohyphal growth. Our results show that there is extensive phenotypic variation between and within strains, and that these six phenotypes are primarily uncorrelated or weakly correlated, with the notable exception of complex colony and complex mat formation. We also show that the phenotypic strength of these strains varies significantly depending on ploidy, and the diploid strains demonstrate both decreased and increased phenotypic strength with respect to their haploid counterparts. This is a more complex view of the impact of ploidy on biofilm-related phenotypes than previous work with laboratory strains has suggested, demonstrating the importance and enormous potential of working with natural isolates of yeast.

Wild-type budding yeast selectively bind to each other and to substrates in cohesive groups called yeast “flocs” (or clumps) and yeast biofilms. The ability of yeast to form biofilms contributes to better survival under stress and affects myriad processes important to human health and industry. In brewing, yeast flocs facilitate easy removal of yeast from the final product, but yeast biofilms can complicate biofuel production and help pathogenic yeast remain virulent on hospital surfaces and during infection (Verstrepen *et al.* 2003, 2004; Bauer *et al.* 2010; Sivakumar *et al.* 2010; Hill *et al.* 2013). In the laboratory, yeast biofilms create significant challenges for many experiments. As a result, biofilm formation has been selected against in most laboratory strains and our knowledge of the genetic basis of yeast biofilm formation remains incomplete.

Yeast flocs and biofilms are two distinct but related phenotypes regulated by environmental changes that cause complex signaling and gene expression responses. A true biofilm requires that yeast both form an extracellular protein matrix and adhere to a surface (Stovicek *et al.* 2010; Vachova *et al.* 2011; Bruckner and Mosch 2012). The matrix is made up of secreted proteins and the biofilm is anchored to surfaces by hydrophobic interactions between yeast cell surface proteins and the surface (Voordeckers *et al.* 2012). Biofilms can be observed phenotypically by several assays including examination of a strain’s ability to adhere to polystyrene surfaces, visualized by staining, a strain’s ability to form a complex colony with an extracellular matrix, and its ability to form a complex mat on low agar media (O’Toole and Kolter 1998; O’Toole *et al.* 2000; Reynolds and Fink 2001; Granek and Magwene 2010; Stovicek *et al.* 2010). Flocs, in contrast to true biofilms, are clumps of yeast adhering to each other by a protein–carbohydrate bond between cell surface proteins and cell wall sugars (Bony *et al.* 1997; Guo *et al.* 2000; Verstrepen *et al.* 2003; Bruckner and Mosch 2012). This phenotype is most commonly observed as clumping and settling in liquid media (Johnston and Reader 1983; Liu *et al.* 1996; Smukalla *et al.* 2008). Due to the intercellular bonds, flocculation is different from the type of clumping that results from mother–daughter cell separation defects. Flocculent clumps have been shown to render the participating yeast cells more resistant to chemical stresses (Smukalla *et al.* 2008). There is also evidence that flocs contain an extracellular matrix, a biofilm-related trait, reflecting the close relationship between biofilm and floc phenotypes (Beauvais *et al.* 2009). The formation of biofilms and flocs is thought to be regulated by similar genes. The most well-studied of these genes are the *FLO* genes, including the important genes *FLO1* and *FLO11*, involved primarily in cell–cell and cell–surface adhesion, respectively, and transcription factor *FLO8*, which regulates expression of the other *FLO* genes (Verstrepen and Klis 2006).

Biofilm-related and flocculation-related phenotypes are predicted to differ depending on ploidy. Another common phenotype associated with *FLO* gene activity is invasiveness into solid agar media, which in haploids occurs on rich media and in diploids occurs under limited nitrogen supply as filamentous growth (Liu *et al.* 1996; Cullen and Sprague 2000; Guo *et al.* 2000; Ryan *et al.* 2012). Diploid strains under a limited nitrogen supply are able to undergo a dimorphic switch from a yeast form, which generates colonies on budding, and the filamentous form, which generates long chains of interconnected cells called “pseudohyphae” that can be seen as filamentous extensions from a colony (Gimeno *et al.* 1992; Liu *et al.* 1996; Lo and Dranginis 1998). Increases in ploidy have also previously been shown to reduce complex colony morphology, complex mat formation, and invasion; this decreased phenotypic strength is thought to result from a cellular response to increased gene dosage or DNA content, or differential expression of cell–surface proteins (Galitski *et al.* 1999; Guo *et al.* 2000; Reynolds and Fink 2001; Granek and Magwene 2010). The possible phenotypic differences between haploid and diploid versions of the same strain and the filamentous growth phenotype specific to diploid strains make a compelling case for considering diploid as well as haploid versions of a strain in any effort to generate a complete picture of its biofilm-forming capabilities.

Natural isolates provide a unique testing ground for understanding the spectrum of biofilm-related phenotypes achieved by *S. cerevisiae* outside of laboratory strains.

The existence of a recent collection of natural yeast isolates from the *Saccharomyces* Genome Resequencing Project (Liti *et al.* 2009) makes it possible to examine important phenotypes that exist among natural yeasts but are not prevalent in laboratory strains. It also provides an opportunity to explore the connection between genotype and biofilm-related phenotypes on an unprecedented scale. The SGRP collection of natural isolates continues to grow, providing an extended resource for examining questions of phenotypic variation in natural isolates and now, with high-quality sequence information available, questions of genotype to phenotype connections (Skelly *et al.* 2013; Bergström *et al.* 2014).

Other recent studies have used the SGRP collection and other similar natural isolate collections [*e.g.*, Phaff Yeast Culture Collection, ARS (NRRL) Culture Collaboration] to examine questions of lineage and sequence similarity across geographical niches (Liti *et al.* 2009; Cromie *et al.* 2013). The SGRP collection also has associated “phenome” data, for which each strain’s morphology, metabolite, gene expression, and protein traits were recorded, as well as the strains’ responses to environmental perturbations, including toxins and nutrient limitations (Warringer *et al.* 2011; Skelly *et al.* 2013; Zorgo *et al.* 2013). With this extensive profile available on the sequence, gene expression, and phenotypic levels, the SGRP collection is a natural and informative starting point for examining genotype/phenotype relationships, and strains from the collection have already been used effectively in a few quantitative trait mapping studies (Cubillos *et al.* 2011, 2013; Granek *et al.* 2013; Jara *et al.* 2014).

Our objective in this study was to assess the diversity of biofilm-related phenotypes in the natural isolate collection and to determine the extent to which any or all of those biofilm-related phenotypes are related. We present a panel of biofilm-related phenotypic assays for 31 haploid and 24 diploid strains in the SGRP natural isolate collection. We show that there is strong qualitative and quantitative variation across six primary phenotypes within the collection: complex colony morphology; complex mat formation; flocculation; agar invasion; polystyrene adhesion; and filamentous growth. The strength of each phenotype is only weakly correlated with the others, with some notable exceptions, demonstrating the potential of these assays to evaluate different aspects of biofilm-related morphology. We also show that the diploid phenotypes are significantly different from their haploid counterparts, in several cases showing increased complexity with respect to the haploid strains contrary to expectations based on the literature about laboratory strains. These findings clearly demonstrate the utility of examining natural isolates to better understand the correlations between these phenotypes and their relationship to ploidy.

## Materials and Methods

### Strains and media used in this study

SGRP strains used in this study (Liti *et al.* 2009) were purchased from the National Collection of Yeast Cultures (https://catalogue.ncyc.co.uk) and are listed in Supporting Information, Table S1. Strain identity and ploidy was verified for a subset of diploid strains by sequencing, sporulation, and cytometry (data not shown). Standard growth medium for plates and liquid overnight cultures was yeast extract peptone dextrose (YPD) media, with 2% glucose and 2% agar for plates. Growth in the polystyrene adherence assay was performed in synthetic complete media with 2% glucose or 0.1% glucose. Standard media were prepared according to Sherman (1998). Diploid filamentous growth was assayed on synthetic low-ammonia histidine dextrose (SLAHD) media prepared according to a modified recipe (Gimeno *et al.* 1992; Lorenz and Heitman 1997) with 50 μM ammonium sulfate, 2% glucose, 2% bacto-agar, 1.7 g YNB without ammonium sulfate or amino acids, and 0.2 mM histidine hydrochloride per liter. Prion curing was conducted on 3-mM guanidine hydrochloride plates prepared according to Holmes *et al.* (2013).

### Standardized phenotypic assay protocols

#### Measuring complex colony morphology:

Two biological replicates of strains were inoculated from colonies into 200 μL YPD liquid overnight cultures in 96-well plates. They were diluted to concentrations of 10^−5^, 10^−6^, and 10^−7^ for haploid strains, with the second biological replicate at concentration 10^−6^ only; 200 μL of all four dilutions of culture was plated using glass beads on 2% agar, 2% glucose YPD plates. The colony plates were incubated at 30° for 3 d and at 25° for an additional 10 d. The colonies were photographed and scored for complexity on days 5, 9, and 13. All imaging in this and other assays was performed using a Canon Powershot SD1200 IS digital camera. This procedure was maintained for all collections (haploid SGRP strains, diploid SGRP strains, and prion-cured strains) with modifications only in the dilutions plated (10^−6^ only for diploid and prion-cured strains). Colony complexity was scored from 0 (smooth colony) to 5 (very strong complex colony morphology) according to the metric described in Table S2, adapted from Granek and Magwene (2010).

#### Measuring complex mat formation:

Strains were inoculated by toothpick from colonies onto the center of a 0.3% agar, 2% glucose YPD plate left to dry for 1 d. The mat formation plates were sealed with parafilm and incubated upright at 25° for 13 d (Reynolds and Fink 2001). On the final day, the complex mats were photographed and scored for complexity according to the metric described in Table S2. This assay was performed once across all strains in the diploid and prion-cured collections and for two biological replicates in the haploid collection. A second biological replicate was performed for eight of the nine diploid strains with phenotypes that differed from the haploid strains. One of these replicates with a weak phenotype was inconclusive by visual scoring.

#### Measuring flocculation as settling in liquid culture:

The settling assay measures clumping and settling of cells to the bottom of 2% glucose YPD liquid culture (Johnston and Reader 1983; Liu *et al.* 1996; Smukalla *et al.* 2008). Strains were inoculated from colonies into 5 mL YPD liquid cultures and grown for 20 hr at 30°. The strains were then vortexed vigorously in the culture tubes and allowed to settle. For each strain, four photographic time points were taken at 0, 15, 30, and 60 min, with the culture tubes undisturbed between time points. This assay was performed once across all strains in the diploid and prion-cured collections and for two biological replicates in the haploid collection.

#### Quantitative flocculation assay:

Quantitative flocculation analysis was conducted on the images from the settled 60-min time point for each strain across two biological replicates of the haploid collection, the diploid collection, and the prion-cured collection. The images were converted to black and white in image processing software Picasa version 3.9.16.37 and analyzed in image processing software ImageJ version 1.47 (Abramoff *et al.* 2004). A line was drawn from the meniscus to the bottom of the culture tube in each image and used to measure the maximum gray intensity and the plot profile of intensity along the line. From the plot profile data, the x-coordinate along the line at which half of the maximum gray was reached was calculated and used to calculate a ratio: x coordinate of half of maximum gray intensity / length of the line. Three of these measurements were taken per image and the average ratio across plot profiles was calculated to determine, for each strain, the percentage of the tube cleared at 60 min.

#### Measuring agar invasion:

The agar invasion assay measures a strain’s ability to invade solid agar media, quantified by the density of cells remaining in the media when colonies are washed off the surface. Strains were inoculated from colonies into 5 mL YPD liquid overnight cultures. They were grown to saturation and standardized to an OD between 0.5 and 0.8. The 2% agar, 2% glucose YPD invasion plates were spotted with all strains from a collection (haploid SGRP strains, diploid SGRP strains, or prion-cured strains) and the FY4 (s288C) reference strain with a 1-μL spot per strain. Four invasion plates were inoculated per collection to provide technical replicates. Invasion plates were grown at 30° for 5 d, after which time the colonies were washed off the surface of the plates under a stream of distilled water using a gloved finger (Liu *et al.* 1996; Roop and Brem 2013). The remaining invaded spots were photographed for each spot on each of the four plates. The washed plates were incubated again at 30° for 24 hr, then washed and photographed again to observe additional growth from cells trapped in the agar (Drees *et al.* 2005; Ryan *et al.* 2012). This assay was performed once across all strains in the diploid and prion-cured collections and for two biological replicates in the haploid collection. Eight diploid strains had a statistically significant difference in invasive ability compared with the haploid strains and these strains were selected for a second biological replicate.

#### Quantitative invasion calculations:

From the four technical replicates of each strain, the three best images were selected for quantitative analysis. The images were converted to black and white in image processing software Picasa version 3.9.16.37 and analyzed in image processing software ImageJ version 1.47 (Abramoff *et al.* 2004). To quantify the invasion for each spot, the mean gray intensity of the spot and surrounding background were calculated in ImageJ. The mean gray intensity of the entire image was also calculated in ImageJ and used to subtract an average background value from the invaded spot. The formula used for background correction calculated the background-corrected intensity value as [(A_t_*I_t_)−(A_s_*I_s_))/(A_t_−A_s_)], where A_t_ is the area of the total image, A_s_ is the area of the invaded spot, I_t_ is the mean gray intensity of the total image, and I_s_ is the mean gray intensity of the invaded spot. For any strains for which background correction yielded a negative invasion value, the value was corrected to zero. This analysis was performed for the full haploid, diploid, and prion-cured collections, and for an additional biological replicate of the haploid collection.

#### Polystyrene adhesion with crystal violet assay:

Polystyrene adhesion is measured by growing biofilms in flat-bottom polystyrene 96-well plates and staining for remaining cells using crystal violet dye. Strains were inoculated into SC cultures with 2% glucose and grown to an OD between 0.5 and 1.5 according to Reynolds and Fink (2001). OD600 was measured on a BioTek Synergy ^1^H hybrid microplate reader. Cells were spun down and washed with distilled water, then resuspended in SC media with 0.1% glucose to a standard OD of 1. Then, 100 μL of culture was inoculated in two technical replicates, three for strains with enough culture volume after resuspension, into a 96-well Costar untreated flat-bottom polystyrene plate. The flat-bottom plate was incubated for 6 hr at 30°. The cultures were then fixed and stained with 100 μL per well of crystal violet dye solution (Fisher Scientific). Crystal violet dye was prepared as a 1% crystal violet solution in 100% ethanol and filter sterilized through a 0.2-μm filter. The crystal violet was incubated in the wells for 15 min at room temperature, then decanted onto absorbent bench pads. The wells were washed three times with 300 μL sterile water, with decanting to rinse. This assay was performed once across all strains in the diploid and prion-cured collections and for two biological replicates in the haploid collection.

#### Quantitative polystyrene adhesion with crystal violet assay:

Stained biofilms were analyzed according to Reynolds and Fink (2001). Biofilms were solubilized in 100 μL 10% SDS per well with a 30-min incubation at room temperature; 100 μL sterile water was then added and 100 μL of solubilized dye was transferred from each well to a new flat-bottom 96-well plate. Absorbance was measured at 570 nm, the absorbance of crystal violet, on a BioTek Synergy ^1^H hybrid microplate reader.

#### Diploid filamentous growth assay:

Strains were plated following the complex colony morphology assay protocol described above onto synthetic low-ammonia histidine dextrose (SLAHD) plates to encourage filamentous growth in a nitrogen-poor environment (Gimeno *et al.* 1992; Liu *et al.* 1996; Lorenz and Heitman 1997). The colony plates were incubated at 30° for 3 d and at 25° for an additional 2 d. The colonies were photographed and scored for filamentous growth under a brightfield microscope at between 20× and 50× magnification. The colonies grew at 25° for an additional 4 d and were photographed and scored for additional filamentous growth on the ninth day of growth. Colonies were scored from 0 to 5 for filamentous growth according to the metric described in Table S2.

#### Curing strains of prions:

To eliminate any prions in the natural isolates, all haploid strains were passaged four times on 3 mM guanidine hydrochloride YPD plates, bottlenecking through a single colony each time (Holmes *et al.* 2013). Clones were selected from the cured strains and passaged on 1.5% agar yeast-extract peptone glycerol media to check for respiration competence. Strains that were respiration competent were stored as prion-negative strains in glycerol stocks; strains that were not respiration-competent were excluded from further experiments. Prion-cured strains were phenotyped according to the same assay protocols described above. For seven strains that showed any potential phenotypic differences after curing and two that did not, additional cured replicates were generated to verify the phenotypes and observe the effects of potential additional mutations resulting from the curing process.

### Correlations between quantitative phenotypes

To determine whether any of the biofilm-related phenotypic traits were correlated, mean quantitative data across each phenotype were compared using a paired two-tailed *t*-test assuming unequal variance. For colony morphology and mat formation, the two qualitative phenotypes scored from 0 to 5, a Kendall’s tau rank correlation test (Kendall 1938) was performed in R on the scores for each strain, comparing each phenotype pairwise.

### Hierarchical clustering of phenotypes by niche

To determine whether any phenotypes clustered by niche of origin, the quantitative phenotypic values for each strain were linearly normalized to a scale of 0 to 5 and used as input into Cluster version 3.0 (De Hoon *et al.* 2004). The strains were clustered hierarchically based on Euclidean distance between the quantitative phenotype vectors and visualized in Java Treeview (Saldanha 2004).

### Statistical comparison of haploid and diploid strains

Quantitative values for haploid and diploid strains across the flocculation, invasion, and crystal violet assays were compared using a paired two-tailed *t*-test assuming unequal variance across all technical replicates for each strain. *P*-values were evaluated for significance using the *q*-values package from Storey with a false discovery rate of 0.05 and the Benjamini-Hochberg method (Storey 2002).

## Results

### Natural isolates exhibit extensive diversity across biofilm-related phenotypes

**Figure 1:**
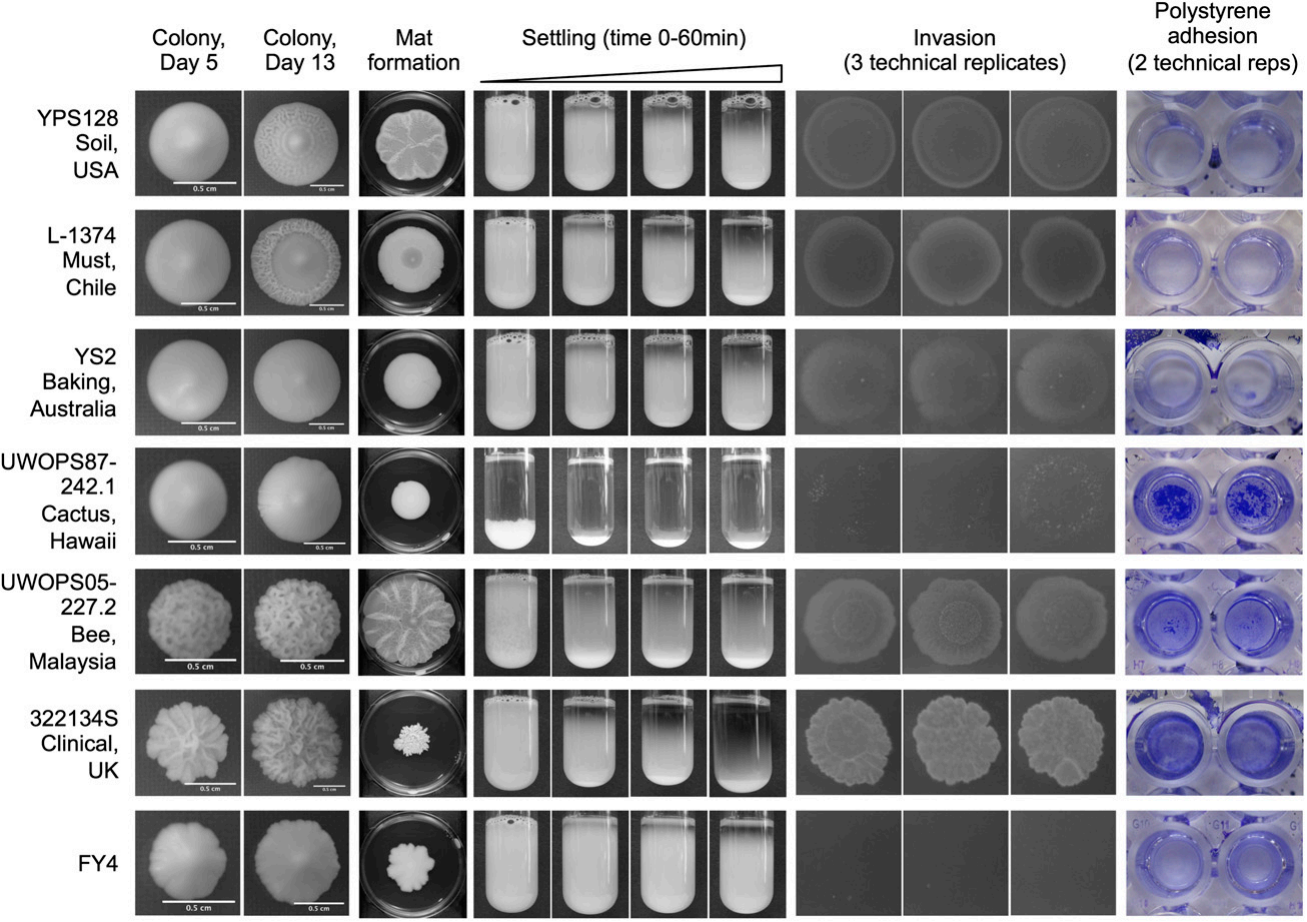
Natural isolates exhibit extensive diversity across biofilm-related phenotypes. Full phenotypic panel for six representative strains and reference strain FY4. Strains are listed with their formal name and origin and are shown across five different phenotypes. Complex colony morphology is shown at day 5 and day 13 of growth on 2% agar YPD plates. Complex mat formation is shown at day 13 of growth on 0.3% agar YPD plates, with the plate included for scale. Settling photo time points were taken at 0, 15, 30, and 60 min. Three technical replicates are shown for the invasion assay and photographed after 24 hr of growth after washing on day 5. Two technical replicates are shown for the polystyrene adhesion assay. Pictured biofilms are fixed and stained with a 1% w/v crystal violet solution.

To determine the diversity of phenotypes present in the *Saccharomyces* Genome Resequencing Project collection (Liti *et al.* 2009), we examined five different biofilm-related phenotypes across 30 MATa haploid strains from the collection and a laboratory strain control (Table S1). The five phenotypes (complex colony formation, complex mat formation, flocculation/settling, agar invasion, and polystyrene adhesion) are shown for a representative set of strains in Figure 1. The full dataset for two biological replicates is provided in File S1. These six representative strains not only originate from varied ecological and geographical niches but also demonstrate the full range of phenotypes we observed in this collection from grade 0 (no phenotype) to grade 5 (very strong phenotype), as outlined in Table S2, with score assignments shown in Table S3. We observed that many strains differed in the strength of each trait. For example, the Hawaiian cactus strain UWOPS87-242.1 exhibits strong flocculation and polystyrene adhesion phenotypes and weak phenotypes across the other assays (Figure 1). Within a single phenotypic grading there was also significant morphological variation making each strain unique. In the complex mat formation assay, for example, strains YPS128, UWOPS05-227.2, and 322134S formed highly complex mats that received qualitative scores of 4 and 5, but all three mats exhibit distinct morphologies (Figure 1). By examining the strains using a range of phenotypic assays, we were able to generate what is essentially a unique profile of biofilm-related phenotypes for each of the 31 strains, showcasing the incredible diversity accessible in this collection of natural isolates.

### Quantitative measures of biofilm-related phenotypes agree with qualitative scoring

In addition to the qualitative scores assigned to each phenotype, the flocculation, invasion, and polystyrene adhesion assays were also amenable to quantitation, as has been described in previous studies (see *Materials and Methods*). Previous literature attempted to assign quantitative metrics to mat formation as well (Reynolds and Fink 2001). Although major mat features were retained across biological replicates in our dataset (Figure S1D), quantifiable features like spoke number and lobe number were not consistent across replicates. In addition, many strains in this collection formed filigreed mats without a spoke/lobe structure (File S1) and would not be quantifiable by those metrics.

**Figure 2:**
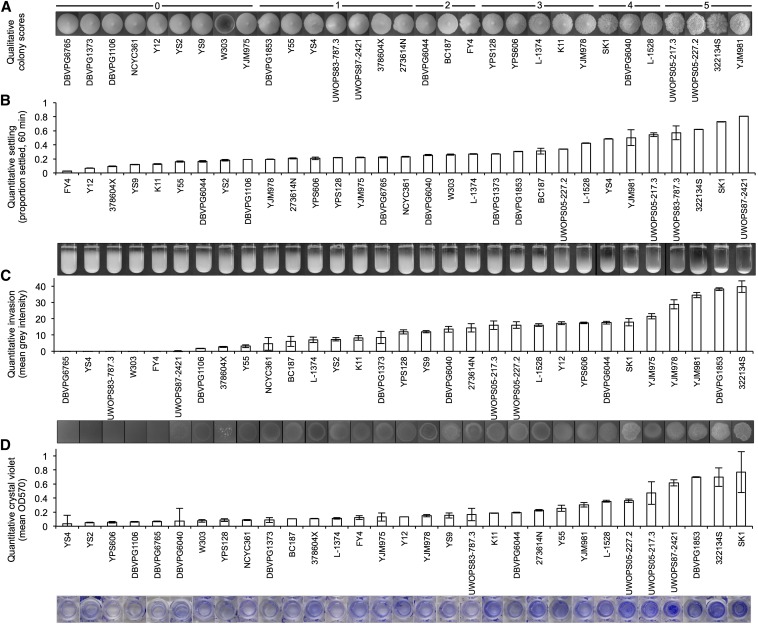
Quantitative measures of biofilm-related phenotypes accurately represent qualitative data. Haploid strains on the x-axis are ranked by increasing phenotypic strength. For procedural details of each method of quantification, see *Materials and Methods*. (A) Qualitative colony classifications at day 13 of growth, from (0) weak to (5) very strong. (B) Quantitative flocculation measured as the percent of culture settled at 60 min. Average of three measurements per image. (C) Quantitative invasion data measured as the mean gray intensity of the invaded spot. Average of three technical replicates. (D) Quantitative crystal violet data measured as OD570 absorbance of crystal violet–stained biofilms. Average of two technical replicates; exceptions are DBVPG6040, YS4 with one measurement only for crystal violet.

The quantitative values across the flocculation, invasion, and polystyrene adhesion assays shown in Figure 2 contrasted with the qualitative image data. Across all three quantitative assays, the strains with the most visually striking phenotypes (UWOPS87-242.1 for settling, 322134S for invasion, and SK1 and 322134S for adhesion) all correspond to the highest recorded quantitative measurements. Distinctions between strains with weak phenotypes are less clearly represented by the quantitative data, but trends of increasing phenotypic strength are consistent across all three quantitative assays. The results of the quantitative assays compared across biological replicates also demonstrate that these assays are a consistent, repeatable method for examining these phenotypes (Figure S1).

### Biofilm-related phenotypes show complex correlations

Our results demonstrate that the phenotypic strength of a strain in one assay is not necessarily predictive of its strength across another. Of the six SGRP strains shown in Figure 1, only two (UWOPS05-227.2 and 322134S) have strong phenotypes across all assays; the rest vary. We were interested in determining if any of these phenotypes show correlations with each other and to what extent each additional assay is providing new information about the behavior of the strain.

**Figure 3:**
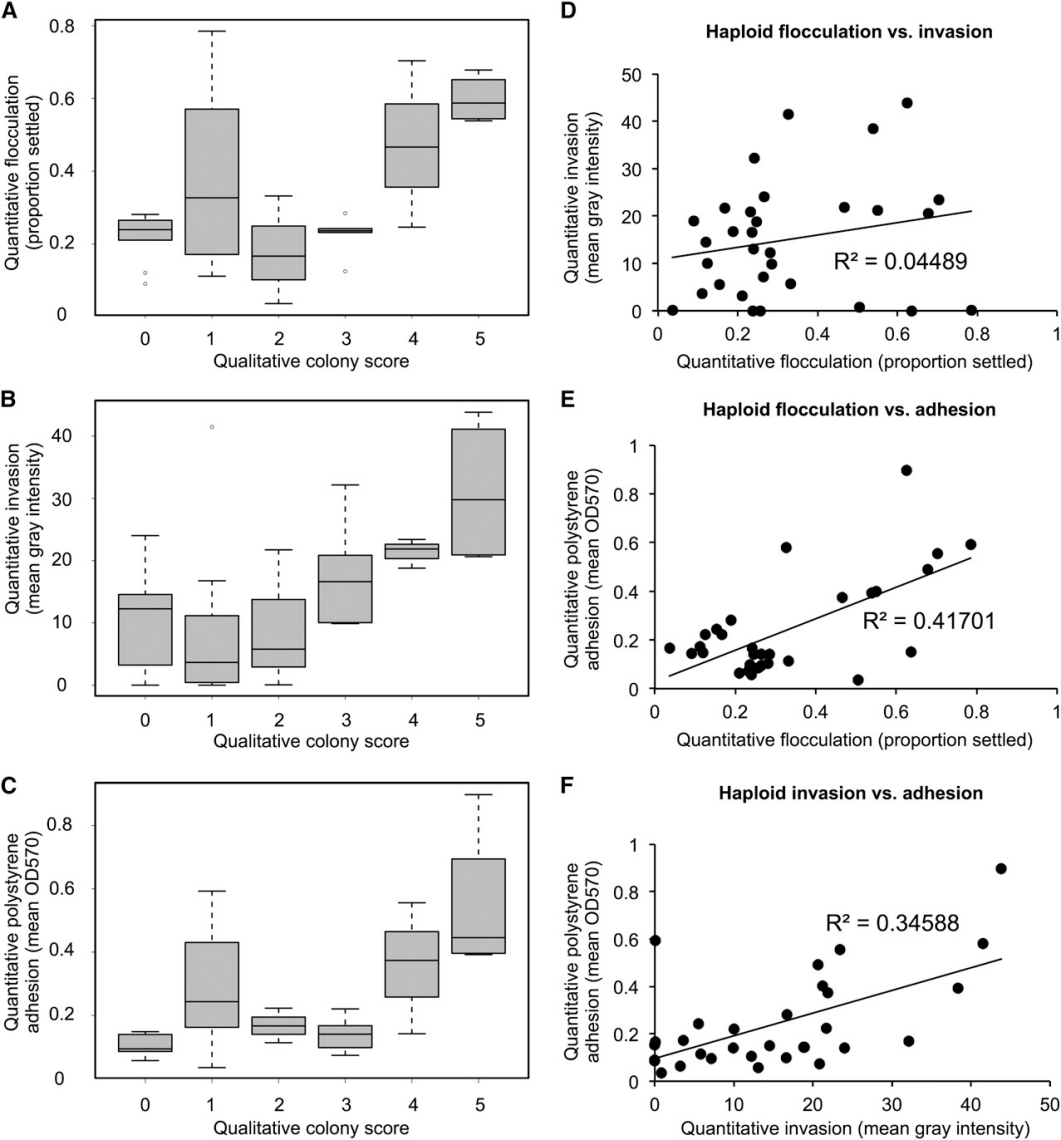
Some biofilm-related phenotypes are correlated. All data are for 31 haploid strains. Qualitative data shown are for a single haploid replicate; quantitative data are averaged across two biological replicates. Mean quantitative data for (A) flocculation, (B) invasion, and (C) adhesion are shown on the y-axis binned according to haploid colony complexity scores (x-axis). Mean quantitative data are plotted for correlation, with (D) flocculation (x) against invasion (y), (E) flocculation (x) against adhesion (y), and (F) invasion (x) against adhesion (y).

For the two assays for which only qualitative data were available (complex colony morphology and complex mat formation), we performed a Kendall’s tau rank correlation test on the qualitative scores across all strains for a single biological replicate in a pairwise fashion. At a significance threshold of *P* < 0.05, we found that these two phenotypes were highly correlated, with a *P*-value of 1.03×10^−6^. For assays with quantitative data, we binned the data across the associated qualitative colony scores (Figure 3, A–C) to look for trends associated with increasing colony morphology complexity. Flocculation (Figure 3A) showed no correlation with colony morphology by this method, but there was a trend associating increasing invasive ability (Figure 3B) and polystyrene adhesion (Figure 3C) with increasing complexity in colony morphology (Figure 3B). For the three assays with quantitative data available, we correlated the average quantitative values across technical replicates between each of the phenotypes. Despite the shared genetic networks implicated in regulation of these traits, the majority of phenotypic correlations we observed were weak. We found no correlation between haploid flocculation and invasion phenotypes (R^2^ = 0.04) (Figure 3D) and a weak correlation between haploid invasion and polystyrene adhesion (R^2^ = 0.35) (Figure 3F). We found a stronger correlation between haploid flocculation and polystyrene adhesion (R^2^ = 0.42) (Figure 3E), although this correlation is still weaker than those between biological replicates (R^2^ = 0.83 and R^2^ = 0.68 from flocculation and adhesion biological replicates, respectively) (Figure S1, B and D).

**Figure 4:**
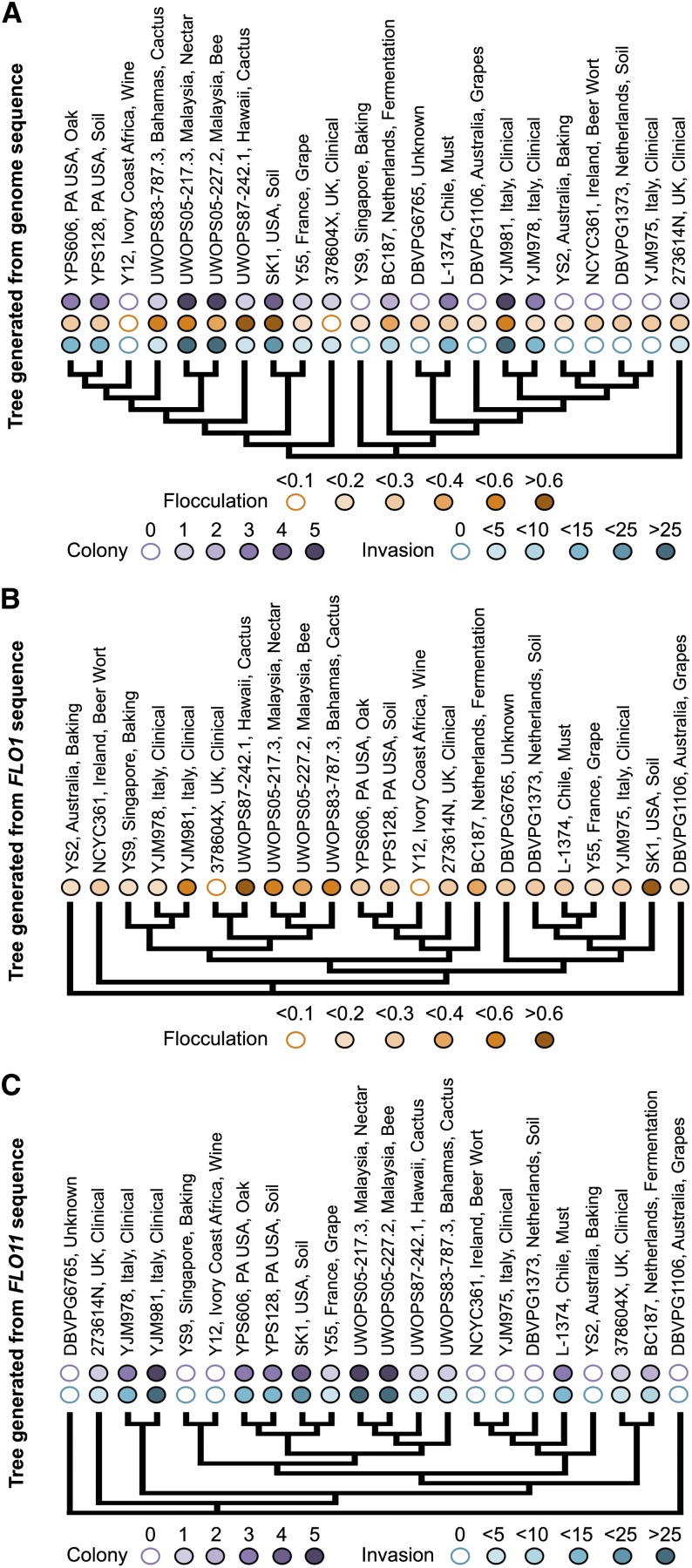
Genetic similarity is not the only predictor of phenotype. (A) 22 SGRP strains clustered by genome sequence in the YRC phenome browser (http://www.yeastrc.org/g2p/). Qualitative and quantitative phenotypic data are shown above each branch, with increasing color intensity corresponding to increasing score. Qualitative colony data are scored and colored from intensity 0 to 5. Quantitative data were binned into 5 corresponding color intensities. (B) Tree generated from *FLO1* gene sequence. Quantitative flocculation data are shown above each branch. (C) Tree generated from *FLO11* gene sequence. Qualitative colony morphology and quantitative invasion data are shown above each branch.

It is noteworthy that these statistical and pairwise comparisons of phenotypes fail to include any phylogenetic information about these strains, and it is possible that some of the weak correlations observed between phenotypes are not representative of true correlations but rather of genetic similarity between the strains being compared. To begin to address this possibility, we examined the distribution of phenotypes across the phylogeny for these strains (Figure 4A) generated in the Yeast Resource Center browser (http://www.yeastrc.org/g2p/). Filled bubbles on each tree represent the phenotypic scores, with a color scale based on the binned quantitative values of the phenotypes. There are examples of genetically similar strains with matching phenotypes (*e.g.*, YPS606 and YPS128, UWOPS05-217.3, and UWOPS05-227.2) but also genetically similar pairs with very different phenotypic signatures (*e.g.*, DBVPG6765 and L-1374).

Because the genome-wide phylogeny of these strains fails to account for the different evolutionary histories of individual genome regions, we also examined these traits at the level of single genes of interest. There are dozens of candidate genes that have been identified for their involvement in biofilm-related phenotypes in QTL studies (Brauer *et al.* 2006; Wilkening *et al.* 2014) and deletion collection studies (Granek and Magwene 2010; Ryan *et al.* 2012). From these identified candidate genes we focused on *FLO1* and *FLO11*, because they have also been extensively shown experimentally to be involved in flocculation (Miki *et al.* 1982; Smukalla *et al.* 2008) and cell–surface interactions (Lo and Dranginis 1996, 1998; Vachova *et al.* 2011), respectively. The gene trees were generated in the Yeast Resource Center phenome project browser using ORF sequences and represent 22 resequenced strains from the collection. In Figure 4B, six of the most flocculent strains are shown juxtaposed on the *FLO1* gene tree; four cluster together while two strains, YJM981 and SK1, are genetically divergent at that locus. This indicates a possible role for certain very similar alleles of *FLO1* in the flocculation phenotype, with YJM981 and SK1 as possible points for comparison. YJM981 also has a different phenotype from its most genetically similar strain at the *FLO1* locus, YJM978. The relationship between *FLO11* genetic variation and phenotype is stronger (Figure 4C). Two phenotypes are shown here, complex colony morphology and invasion, both of which are known to be associated with *FLO11* expression (Galitski *et al.* 1999; Granek and Magwene 2010). There are several instances of genetically similar pairs with identical or similar phenotypes (YS9 and Y12, YPS606 and YPS128, UWOPS05-217.3 and UWOPS05-227.2, NCYC361 and YJM975, 376804X and BC187), and instances of strains similarly related genetically at this locus with different phenotypes show weak differences only (SK1 and Y55, YJM978 and YJM981). To effectively understand the role of genetic variation in these biofilm-related phenotypes, a more targeted approach might be successful.

### Phenotypic pattern does not cluster according to environmental or geographical niche

**Figure 5:**
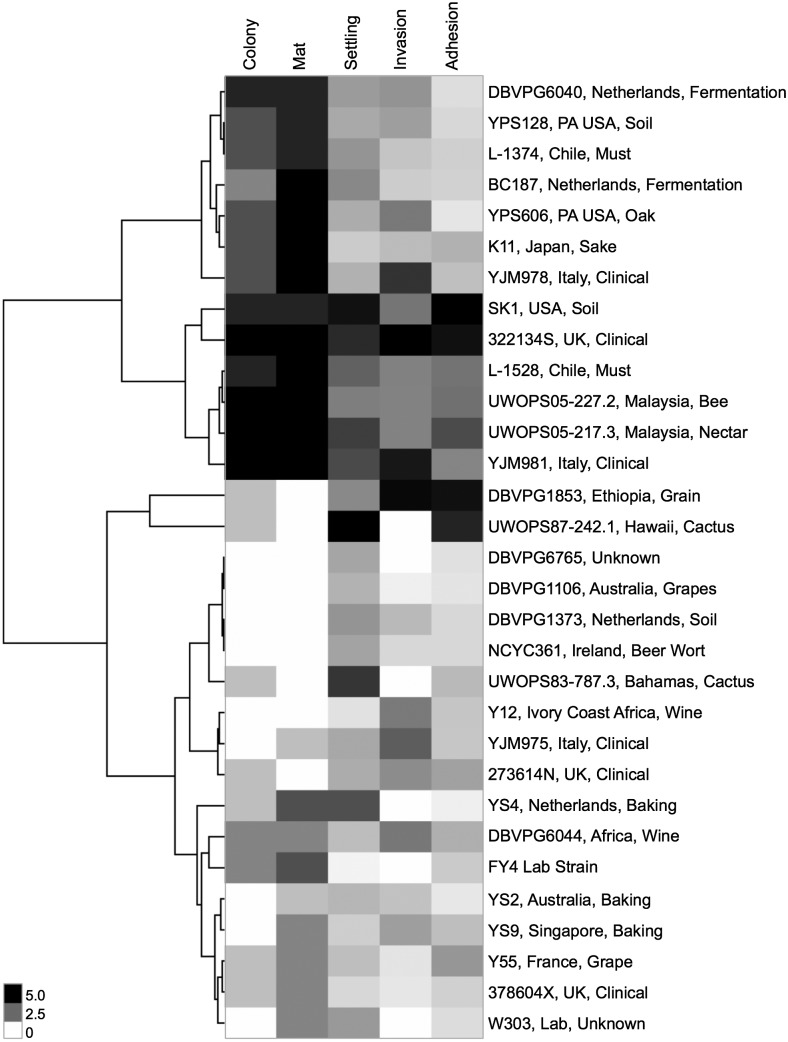
Few strains cluster phenotypically according to niche of origin. Hierarchical clustering of qualitative phenotypes (scores 0 to 5) and quantitative phenotypes (linear normalization to scale 0 to 5) for 31 haploid strains with simplified niche labels according to Liti *et al.* (2009). Cluster 3.0 (De Hoon *et al.* 2004) generated dendrogram based on Euclidean distance between strains; clustering visualized in JavaTreeView (Saldanha 2004). No phenotype (0) registers as white; very strong phenotype (5) registers as black.

To determine if any of the phenotypic fingerprints of these strains clustered by their niche of origin, we examined the hierarchical clustering of the qualitative and quantitative scores by Euclidean distance using the simplified designations for each strain provided by Liti *et al.* (2009). In Figure 5, two primary clusters emerge with strong phenotypes, with the cluster including strains DBVPG6044 through YJM978 driven by strong colony morphology and mat complexity, and the cluster including strains SK1 through YJM981 with strong phenotypes across all assays. No strong clusters emerge for the rest of the strains with weaker phenotypes. Some strains with similar origin niches do cluster together, including Pennsylvania strains YPS128 and YPS606 in the top cluster and Malaysian strains UWOPS05-217.3 and UWOPS05-227.2 in the second cluster. Both of these pairs also have a high degree of sequence similarity, shown through their relatedness at both the *FLO1* and *FLO11* loci and genome-wide in Figure 4. However, there are also several exceptions where strains with very similar niches do not cluster together. Examples include the five clinical strains, cactus strains UWOPS87-242.1 and UWOPS83-787.3, and Chilean must strains L-1374 and L-1528, which do not cluster together phenotypically despite sharing the same ecological and geographical niches.

### Colony morphology and agar invasion phenotypes in some natural isolates might be driven by prion content

We hypothesized that nongenetic causes may affect some of the correlations and lack thereof that we observed. Previous work on natural isolates has shown that up to 8% of natural isolates may harbor prions (Halfmann *et al.* 2012). Some of the phenotypic changes in natural isolates cured for prions include differences in colony morphology, invasion, and flocculation phenotypes (Holmes *et al.* 2013). To determine if the biofilm-related phenotypes in these natural isolates were driven by prions, we cured the strains of prions according to the procedure outlined by Holmes *et al.* (2013) (see *Materials and Methods*). We tested strains after passaging for respiration competence on glycerol plates and six strains repeatedly failed to be respiration competent after curing. For the remaining 25 cured strains, we completed a full phenotypic panel across four assays (File S2), excluding complex mat formation because of its strong correlation with complex colony morphology. For nine of those strains, we also generated an additional, separately cured replicate to confirm the cured phenotype; these replicates are shown in File S2 as well. We observed no significant phenotypic differences from the haploid strains in our cured strains across all phenotypes except colony morphology and agar invasion (Figure S2). Three strains (273614N, YJM978, and YPS128) showed a reduction or variation in complex colony morphology after curing. Two additional cured strains (DBVPG1853 and W303) exhibited changes in colony morphology that were not carried through all cured replicates. These five strains had little shared history, spanning four ecological and five geographical niches. The agar invasion phenotype has both a characteristic intensity and a characteristic invasion pattern for each strain that is highly reproducible across biological replicates (File S1). For strains YPS128, DBVPG1106, Y12, YS2, YS4, and YS9, the prion-cured strains exhibited changes in both invasion intensity and pattern; Y12 also demonstrated differences between cured replicates. These differences are reflected in the correlation plot for haploid *vs.* prion-cured strains across the invasion phenotype (Figure S2B) with an R^2^ value of 0.83 lower than the haploid biological replicates (R^2^ = 0.95). The six strains with altered invasion phenotypes in their prion-cured versions fall across four ecological niches and five geographical niches; all of the baker strains (YS2, YS4, YS9) and all of the Australian strains (DBVPG1106, YS2) in the collection potentially harbor prions.

### Diploid strains show changes in correlations between biofilm-related phenotypes

To determine how ploidy may affect these traits, we completed a full phenotypic panel of all six assays, including complex mat formation, on the homothallic diploid SGRP collection. The complete panel is shown in File S4. We also examined an additional phenotype, filamentous growth, which is specific to the diploid strains (Gimeno *et al.* 1992; Liu *et al.* 1996). We binned the quantitative invasive growth scores into five groups according to the same metric used in Figure 4 and compared those scores with the qualitative filamentous growth scores with a Kendall’s tau rank correlation test. These two phenotypes are significantly correlated, with a *P*-value of 0.04. We repeated the low-nitrogen filamentous growth assay for the haploid collection as a control to ensure that none of the haploid strains demonstrated a filamentous growth phenotype. We observed no filamentous growth for any haploid strains (data not shown).

**Figure 6:**
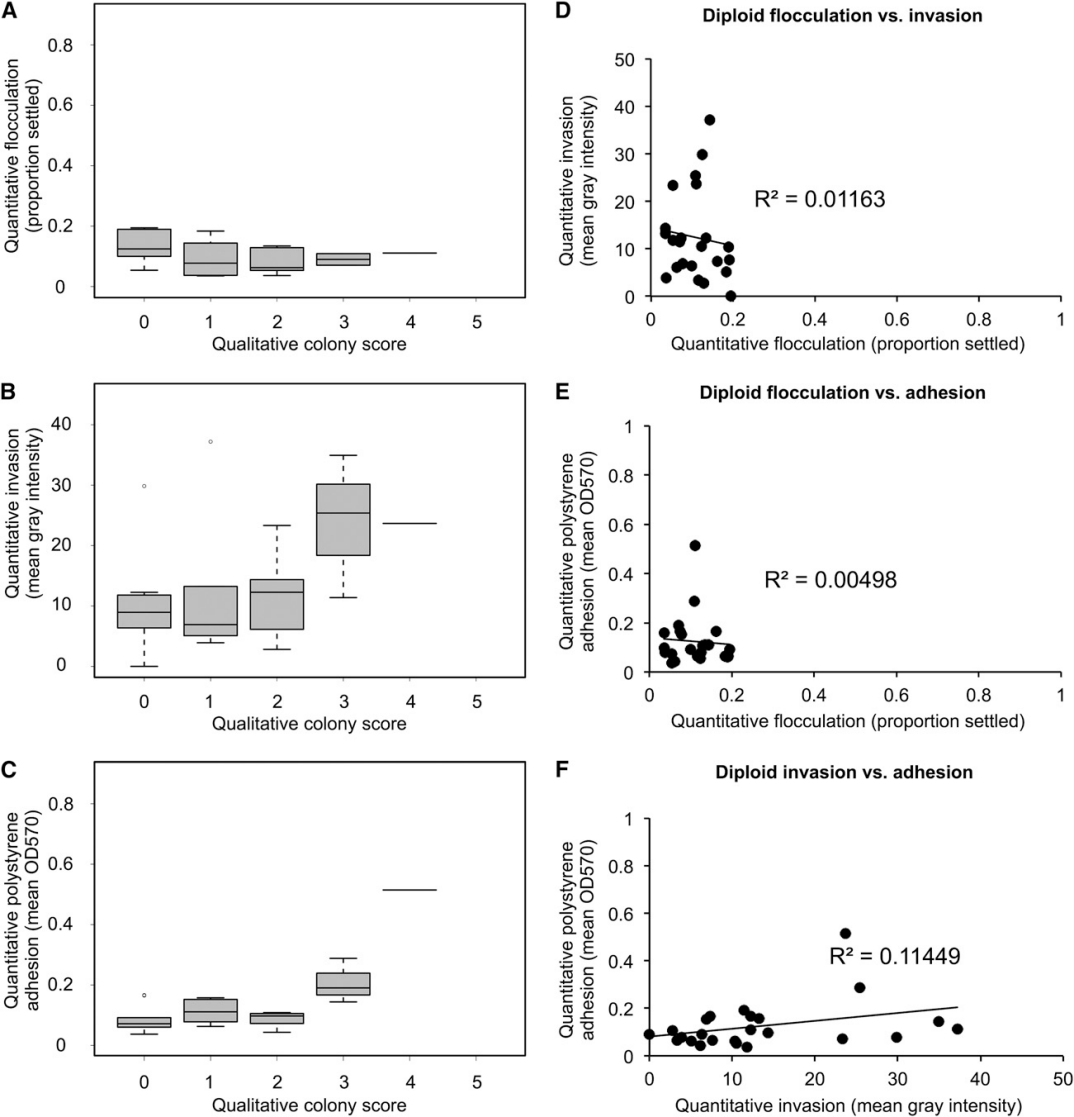
Diploid strains show weaker correlations between biofilm-related phenotypes. All data are for 24 diploid strains and averaged across technical replicates (see *Materials and Methods*). Mean quantitative data for (A) flocculation, (B) invasion, and (C) adhesion are shown on the y-axis binned according to diploid colony complexity scores (x-axis). Mean quantitative data are plotted for correlation, with (D) flocculation (x) against invasion (y), (E) flocculation (x) against adhesion (y), and (F) invasion (x) against adhesion (y).

We repeated the correlation analyses we performed on the diploid collection to observe how the relationships between traits change in the diploids. We compared the quantitative diploid data across the three quantitative traits (flocculation, invasion, adhesion) with the complex colony morphology scores and observed a weak trend of increasing colony morphology associated with increased invasion (Figure 6B), similar to the haploids. We also performed a Kendall’s tau rank correlation test on the diploid qualitative data; we found that the relationship between the colony morphology and mat formation assays changed with the change in ploidy, with the previous correlation lost (new *P* = 0.08, above the significance threshold *P* < 0.05). Several other correlations are lost in the diploid data, as shown in Figure 6. There is no correlation between any of the quantitative phenotypes (Figure 6, D–F) with the correlation between flocculation and adhesion we observed in the haploids specifically lost in the diploids (R^2^ = 0.005) (Figure 6E).

### Diploid strains do not uniformly have reduced phenotypic strength with respect to haploid strains

The strength and complexity of several of these biofilm-related phenotypes have been reported to be influenced by ploidy. Galitski *et al.* (1999) and Reynolds and Fink (2001) observed that an increase in ploidy in laboratory strain Σ1278B results in both decreased invasive ability on agar as well as decreased mat complexity. A correlation between increased ploidy and decreased complex colony morphology across natural isolates has also been reported (Granek and Magwene 2010).

**Figure 7:**
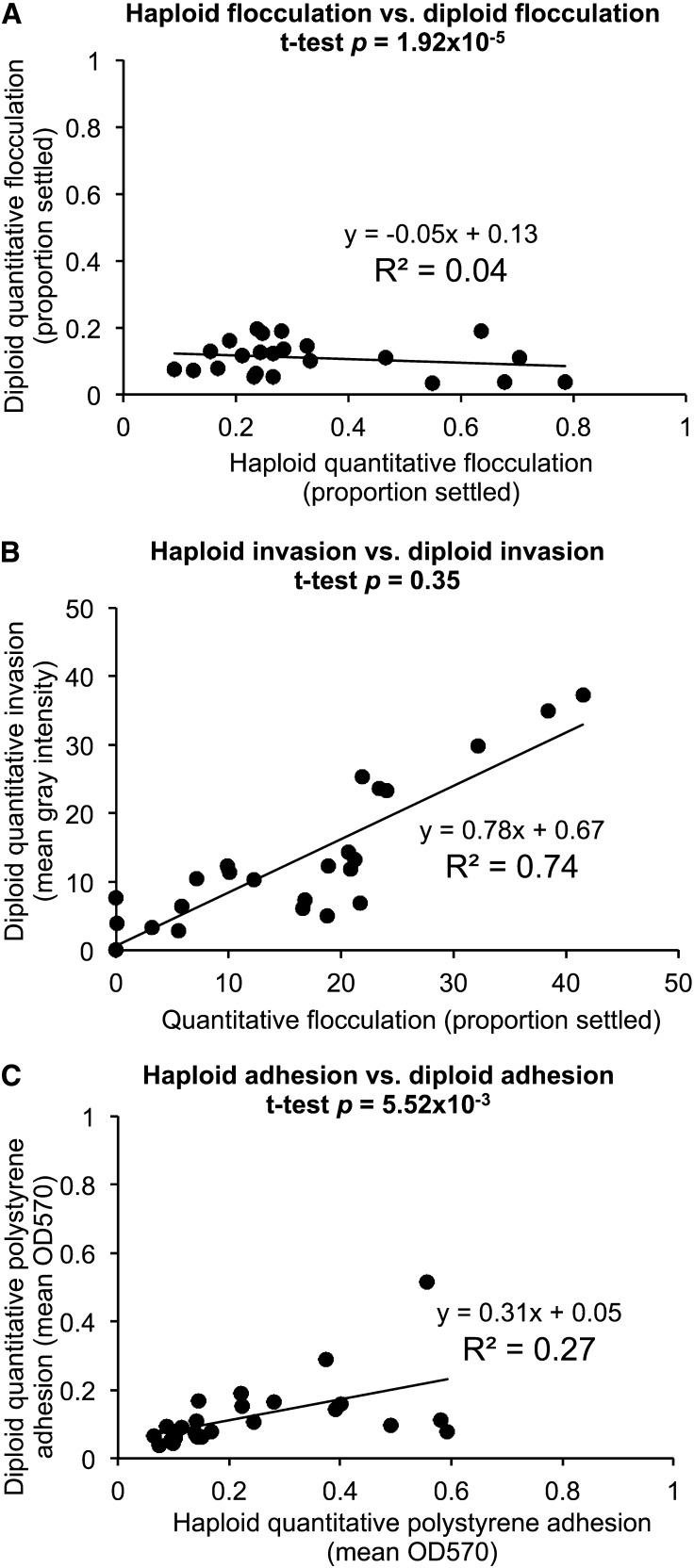
Diploid quantitative phenotypes are weaker than haploid quantitative phenotypes except for invasion. (A) Mean quantitative haploid flocculation data for two biological replicates (x-axis) are plotted against quantitative diploid flocculation data (y-axis) for correlation. (B) Mean quantitative haploid invasion data for two biological replicates (x-axis) are plotted against quantitative diploid invasion data (y-axis) for correlation. (C) Mean quantitative haploid polystyrene adhesion data for two biological replicates (x-axis) are plotted against quantitative diploid polystyrene adhesion data (y-axis) for correlation. All quantitative haploid and diploid data are available in File S3.

**Figure 8:**
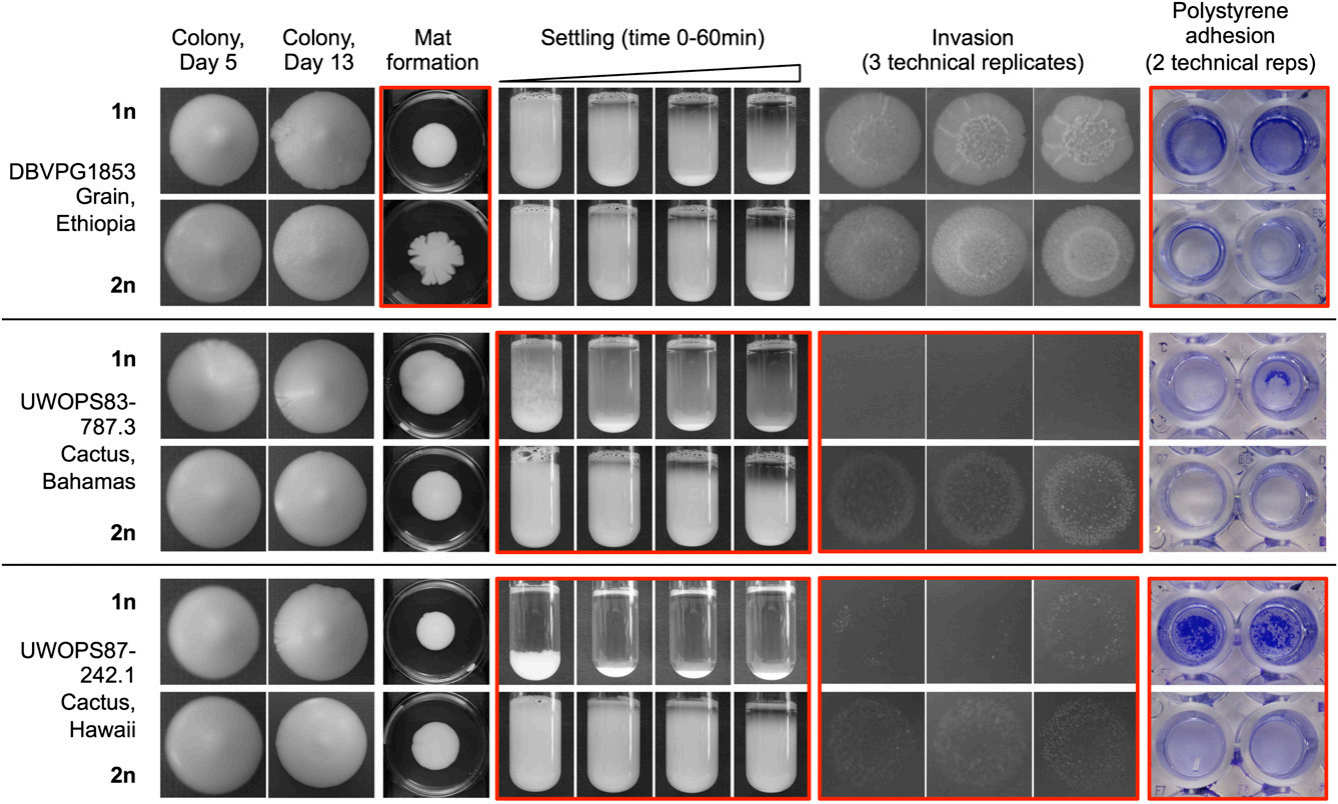
Examples of phenotypic differences depending on ploidy. Three representative strains are listed with their formal name and origin and are shown across five different phenotypes, with ploidy designated “1n” or “2n” for haploid and diploid strains. Red boxes indicate notable changes in phenotype between haploid and diploid versions of each representative strain. Image details and experimental methods are the same as Figure 1.

Across the five primary phenotypic assays, we observed that the strength of the phenotypes differed with respect to the haploid strains and were generally attenuated across all phenotypes (Figure 7). No diploid strains achieved a complex colony morphology score of 5 (Figure 6, A–C), a complex mat score of 5, or a flocculation score of more than 0.3, where the highest haploid flocculation score was 0.8 (strain UWOPS83-787.3) and 16 strains achieved a score of 0.3 or higher. These trends are reflected in the haploid/diploid correlation plots in Figure 7. In Figure 7A and Figure 7C, the low slopes (m = −0.05 and m = 0.31, respectively) and correlation coefficients (R^2^ = 0.04 and R^2^ = 0.27) demonstrate that in the flocculation and polystyrene adhesion assays, diploid strains have very different phenotypes from their haploid counterparts and these phenotypes are weaker than the haploid phenotypes. These numbers are particularly striking compared with the haploid/prion-cured differences, which should be negligible if the phenotypes are not strongly influenced by prions. In fact, we do find that to be the case with 20 out of 26, 20 out of 23, and 23 out of 25 prion-cured strains showing no significant change in flocculation, invasion, and adhesion, respectively, compared with the haploids (Table S4). These values confirm that the magnitude of the changes in the diploid strains is well outside the range of biological noise in the assay. The invasion assay correlation, shown in Figure 7B, shows a different pattern. The high R^2^ value of 0.74 is slightly lower than the biological replicate noise level (R^2^ of 0.95 for invasion) (Figure S1C) and the slope m = 0.77 close to 1 indicates that the haploid and diploid quantitative invasion values are very similar. Contrary to predictions from literature, we observe two cases of increases in invasive ability in the diploid strains (shown in Figure 6 and Table 1). This increase in invasive ability was statistically significant in a *q*-value test between average haploid and diploid values for strains UWOPS87-242.1 and UWOPS83-787.3. This increase in invasive ability is also visible in the invasion images (Figure 8).

**Table 1:** Quantitative changes in diploid strains *vs.* haploid strains

	Flocculation*^a^*	Invasion*^b^*	Adhesion*^c^*
Significant decrease by FDR correction*^d^*	22	6	11
No change by FDR correction	1	15	13
Significant increase by FDR correction	0	2	0
	**Colony**	**Mat**	
Decrease by qualitative score	13	12	
No change by qualitative score	9	3	
Increase by qualitative score	2	9	

The numbers of diploid strains that showed differences when compared with the related haploid strains across three quantitative metrics are shown. Quantitative values were compared using a paired two-tailed *t*-test assuming unequal variance across all technical replicates for each strain. *P*-values were evaluated for significance using the *q*-values package from Storey with the Benjamini-Hochberg method (Storey 2002).

a 23 strains.

b 23 strains.

c 24 strains.

d FDR = 0.05.

Although we saw examples of diploid strains with weakened phenotypes across all metrics, there were several notable counterexamples. In Figure 8, weakened phenotypes are shown for strains DBVPG1853 and UWOPS87-242.1, which lose their polystyrene adhesive ability as diploids, and strains UWOPS83-787.3 and UWOPS87-242.1, which both lose their strong flocculation ability as diploids. However, some exceptions emerge: strain DBVPG1853 gains a complex mat as a diploid, and invasive ability improves in the diploid versions of UWOPS83-787.3 and UWOPS87-242.1, especially for UWOPS83-787.3. Across qualitative scores for a single haploid biological replicate, nine strains showed a more complex mat as diploids than as haploids, although in five of these cases the qualitative scores show a weak gain of complexity only (Table S3). The exceptions in the quantitative assays are also highlighted in a statistical comparison of the haploid and diploid data shown in Table 1, which shows the number of diploid strains that showed a significant increase or decrease across each phenotypic category with respect to the haploid strains (see *Materials and Methods*). With those conditions applied, 22 of 23 tested strains had a significant decrease in flocculation as diploids, but only six strains had a significant decrease in invasive ability. The most common scenario was to see no significant difference between haploids and diploids (15 out of 23 strains for invasion, 13 out of 24 strains for adhesion). Instances of no significant difference were not dominated by strains with weak phenotypes; of the 15 strains with no significant difference in invasive ability, only six strains had diploid mean gray intensity values less than 10.

## Discussion

Understanding connections between genotype and phenotype is one of the primary driving motivations behind biology and genomics today, yet many of the tools we are using to answer this question are constrained by lack of phenotypic diversity. Biofilm-related phenotypes in yeast, for example, are limited in laboratory strains and only now with natural yeast isolates are we able to examine the full diversity of phenotypes and their possible genetic origins. In this study we have demonstrated the incredible amount of phenotypic diversity present across biofilm-related phenotypes in just 30 natural isolates, a limited panel. With this phenotypic panel, we have shown that despite the predicted involvement of similar genetic pathways, many of these biofilm-related phenotypes are uncorrelated or weakly correlated with each other and are potentially controlled by different genetic networks. We have also demonstrated that the majority of phenotypes we observe are not due to prions and are variable depending on ploidy, but not in predictable ways. General conclusions about the effects of ploidy on biofilm-related phenotypes have been drawn in the literature based on only a few strain backgrounds and, although these trends are generally observed in our data, there are also notable exceptions. These findings demonstrate the importance of using natural isolates to understand genotype–phenotype relationships because many predictions made in laboratory strains do not hold true for natural isolates.

In comparing complex colony morphology, complex mat formation, flocculation/settling, agar invasion, and polystyrene adhesion/crystal violet phenotypes in haploid natural isolates, we found two strongly correlated relationships (complex colony morphology and complex mat formation; flocculation and polystyrene adhesion) and two weakly correlated relationships (complex colony morphology and invasion; invasion and polystyrene adhesion). Given that most of the relationships we observed in Figure 3 were weakly correlated or uncorrelated, this suggests complex genetic interactions underlying each phenotype, although some of the stronger correlations could be explained by shared genetic drivers. All of these traits have been shown in previous work to be dependent on *FLO11* activity (Lo and Dranginis 1998; Reynolds and Fink 2001; Granek *et al.* 2013), including flocculation under specific conditions (Bayly *et al.* 2005), although flocculation is primarily considered to be a *FLO1*-mediated trait (Smukalla *et al.* 2008). This difference in genetic control is potentially responsible for the lack of correlation we observed between quantitative flocculation and invasion values (R^2^ = 0.03), which is contrary to the correlation between these phenotypes observed in *S. paradoxus* (Roop and Brem 2013). It would have been equally unsurprising to see a lack of correlation between the flocculation and polystyrene adhesion assays, but it was not what we observed in the quantitative data (R^2^ = 0.47); in fact, it was the strongest correlation we observed. The caveat to this relationship is that we observed that clumpy strains are less likely to form a consistent monolayer of cells in the polystyrene adhesion assay, instead retaining some of their clumpiness in the biofilm. These clumps absorb more of the crystal violet stain, potentially skewing the adhesion results in the direction of particularly flocculent strains. This could potentially be addressed in the future by treating the cultures with a deflocculation buffer prior to either biofilm formation or staining. More interesting is the weak correlation between the invasion phenotype and the polystyrene sticking phenotype (R^2^ = 0.29), as previous studies have shown that *FLO11* is involved in the manifestation of both phenotypes. This suggests that though these phenotypes have a connection to *FLO11* activity, they are likely regulated by more complex genetic interactions.

Several of these phenotypes in the *S. cerevisiae* Σ1278B knockout collection were recently examined (Ryan *et al.* 2012) and it was found that, with a few exceptions, different “growth programs” like filamentous growth and mat formation were controlled by many different genes in an only semi-overlapping fashion. Given that we observed only one very strong correlation between biofilm-related phenotypes (complex colony morphology and complex mat formation), this compartmentalized management of growth programs is what our data suggest as well. The practical outcome of these findings is that to consider the biofilm-related phenotype of one of these natural isolate strains fully, many different assays are needed to accurately describe the characteristics of the strain. The independence of these phenotypes further provides future opportunities to differentiate and classify the genes and pathways affecting each biofilm-related phenotype.

We hypothesized that diploid phenotypes would be weaker in general than the haploid phenotypes for the corresponding strains, according to the work of Galitski *et al.* (1999), Reynolds and Fink (2001), and Granek and Magwene (2010), who each showed that strength of invasion, strength of complex mat formation, and strength of complex colony morphology decrease with ploidy. We observed this same general trend across all phenotypes with some interesting exceptions; across natural isolates, the relationship between the haploid and diploid phenotypes varied according to strain and phenotype examined. Within our representative set of strains in Figure 8, there are examples of phenotypes both stronger and weaker in the diploid strains, which is a characteristic of the collection as a whole (see haploid strains in File S1 and diploid strains in File S4). Most importantly, we have recorded examples in this dataset of significantly increased phenotypic strength in the diploid strains across the complex mat formation and invasion assays; both assays had an attenuated diploid phenotype that was predicted based on experiments in laboratory strains. Our finding that predictions about the effect of ploidy on biofilm-related phenotypes are not necessarily borne out in natural isolates is consistent with previous findings that were discovered on examining ploidy–environment interactions for SGRP haploid and diploid natural isolates, and countless exceptions to predictions of how ploidy would impact a strain’s response to environmental perturbations were also discovered (Zorgo *et al.* 2013). Other studies with this collection suggest laboratory strain S288C is a phenotypic outlier with respect to natural isolates (Warringer *et al.* 2011), a further reminder that the phenotypes recorded from laboratory strains of *S. cerevisiae* are not necessarily generalizable to natural isolates. The SGRP collection and other collections of natural isolates will allow us to test predictions generated from analyzing laboratory strains and to see under what conditions those predictions hold true.

Given a larger subset of strains, the conclusions we draw about the correlations between these phenotypes would undoubtedly be refined. With a sample set of more than 30 strains, the data generated about phenotypic relationships and especially niche relationships would particularly benefit; for some of the niches in which we observed clustering (Figure 5), there were only a few representatives from a given niche (*e.g.*, only three African strains, only three baking strains, only two Malaysian strains). As a result, the conclusions we draw come with a significant caveat. Although our clustering analysis is likely underpowered, the lack of correlation we saw between strength of biofilm-related traits and any particular niche is consistent with observations from Warringer *et al.* (2011), who demonstrated that trait variation across an enormous panel of phenotypes was not driven primarily by the niche of origin.

With a larger collection it would also be critical to develop high-throughput and potentially automated versions of the standard phenotypic assays described in this work. We could potentially refine the picture further by expanding the phenotypic assays to examine natural isolates’ behavior under nutrient-limited conditions, which have been shown to alter biofilm-related phenotypes (Palecek *et al.* 2002; Granek and Magwene 2010). There is also room to improve the scope of the quantitative assays. Right now, the assays only capture one primary dimension of each phenotype, but there is additional variation that stratifies the phenotypes even further. For example, in the flocculation assay plot profiles as shown in Figure S3 and Figure S4, there is additional information about the way a strain settles in the slope and integral of the plot profile line that is not captured by taking a single distance measurement. Similarly, in the invasion assay as shown ranked in Figure 2B, strains with very similar quantitative invasion scores have clearly different invasion structures, with the majority of the intensity driven by a strong edge (*e.g.*, L-1528 and YS9), a punctate pattern (*e.g.*, 378604X), or a completely invaded patch (*e.g.*, Y12 and K11); this fine-scale information is lost in the current invasion quantification metric. There is also room to generate improved quantitative data across the two assays that remain qualitative in this study, particularly the complex colony morphology assay. Quantitative methods for measuring complex colony morphology are becoming available using time-course imaging and specialized image analysis software (Ruusuvuori *et al.* 2014). In future work with natural isolates, it would be useful to assign quantitative scores to the complex colonies in this natural isolate collection as well.

We anticipate that the phenotypic data generated in this study will be a valuable platform for examining genotype–phenotype questions in a targeted way. Now that we appreciate the complexity of biofilm-related phenotypes across these strains, we plan to examine the contributions of variation in individual alleles to each phenotype in the context of different genetic backgrounds. We also hope that the phenotypic data generated in this study will be a useful community resource for the many groups now working with strains from this collection. Ultimately, understanding the contributions of variation in known biofilm-related genes to each of these phenotypes could also contribute to effective engineering of strains with very specific biofilm-related traits.

## Supplementary Material

Supporting Information
