# Supplementary material for: Ploidy-Regulated Variation in Biofilm-Related Phenotypes in Natural Isolates of Saccharomyces cerevisiae
Source: G3 (Bethesda). 2014 Jul 24;4(9):1773–86. doi: 10.1534/g3.114.013250 (PMC4169170; doi:10.1534/g3.114.013250)
Supplement: Supporting Information [file supp_4_9_1773__index.html]

Ploidy-Regulated Variation in Biofilm-Related Phenotypes in Natural Isolates of Saccharomyces cerevisiae — Supporting Information 

# Ploidy-Regulated Variation in Biofilm-Related Phenotypes in Natural Isolates of *Saccharomyces cerevisiae*

## Supporting Information for Hope and Dunham, 2014

**Files in this Data Supplement:**

- Supporting Information - Figures S1-S4, Files S1-S4, and Tables S1-S4 (PDF, 746 KB)
- Figure S1 - Haploid biological replicates demonstrate consistency of quantitative assays. (PDF, 403 KB)
- Figure S2 - Prion-cured vs. haploid correlation plots show reproducibility. (PDF, 391 KB)
- Figure S3 - Haploid settling plot profiles. (PDF, 588 KB)
- Figure S4 - Diploid settling plot profiles. (PDF, 487 KB)
- File S1 - Complete haploid phenotype dataset. (PDF, 4 MB)
- File S2 - Complete prion‐cured phenotype dataset. (PDF, 3 MB)
- File S4 - Complete diploid phenotype dataset. (PDF, 3 MB)
- Table S1 - *S. cerevisiae* strains included in this study. (PDF, 144 KB)
- Table S2 - Qualitative scoring metrics. (PDF, 82 KB)
- Table S3 - Qualitative score assignments. (PDF, 82 KB)
- Table S4 - Quantitative changes in prion‐cured strains vs. haploid strains. (PDF, 112 KB)
- File S3 - Table of quantitative assay results. (.txt, 5 KB)
