# Supplementary material for: Ploidy-Regulated Variation in Biofilm-Related Phenotypes in Natural Isolates of Saccharomyces cerevisiae
Source: G3 (Bethesda). 2014 Jul 24;4(9):1773–86. doi: 10.1534/g3.114.013250 (PMC4169170; doi:10.1534/g3.114.013250)
Supplement: Supporting Information [file supp_g3.114.013250_013250SI.pdf]

G3·Genes | Genomes | Genetics

Ploidy-regulated variation in biofilm-related phenotypes in natural isolates of *Saccharomyces cerevisiae*

Elyse A. Hope and Maitreya J. Dunham[1]

Department of Genome Sciences, University of Washington, Seattle, WA 98103

[1]Corresponding author: Foege Building S403B, Box 355065, 3720 15th Ave NE, Seattle, WA 98195, USA. E-mail: maitreya@uw.edu

DOI: 10.1534/g3.114.013250

Table S1 ***S. cerevisiae*** **strains included in this study**

NAME	GEOGRAPHICAL ORIGIN	ECOLOGICAL ORIGIN	MATING TYPE	DIPLOID INCLUDED
DBVPG6765	Unknown	Unknown	a	yes
SK1	USA	Soil	a	yes
DBVPG6044	West Africa	Bili wine, from *Osbeckia grandiflora*	a	yes
DBVPG1373	Netherland	Soil	a	yes
DBVPG1853	Ethiopia	White Teff	a	yes
Y55	France	Grape	a	yes
YPS128	Pennsylvania, USA	Soil beneath *Q. alba*	a	yes
DBVPG1106	Australia	Grapes	a	yes
DBVPG6040	Netherland	Fermenting fruit juice	a	yes
BC187	Napa Valley, USA	Barrel fermentation	a	yes
YPS606	Pennsylvania, USA	Bark of *Q. rubra*	a	yes
L-1374	Chile	Fermentation from must Pais	a	yes
L-1528	Chile	Fermentation from must Cabernet	a	yes
NCYC361	Ireland	Beer spoilage strain from wort	a	yes
K11	Japan	Shochu sake strain	a	yes
Y12	Africa - Ivory Coast	Palm wine strain	a	yes
YS2	Australia	Baker strain	a	no
YS4	Netherlands	Baker strain	a	no
YS9	Singapore	Baker strain	a	no
UWOPS83-787.3	Bahamas	Fruit, *Opuntia stricta*	a	yes
UWOPS87-2421	Hawaii	Cladode, *Opuntia megacantha*	a	yes
UWOPS05-217.3	Malaysia	Nectar, Bertram palm	a	yes
UWOPS05-227.2	Malaysia	*Trigona* spp (Stingless bee) collected near Bertram palm flower	a	yes
W303	Unknown	N/A	a	no
322134S	RVI, Newcastle, UK	Clinical isolate (throat sputum)	a	no
378604X	RVI, Newcastle, UK	Clinical isolate (sputum)	a	no
273614N	RVI, Newcastle, UK	Clinical isolate (fecal)	a	yes

YJM978	Bergamo, Italy	Clinical isolate (vaginitis)	a	yes
YJM981	Bergamo, Italy	Clinical isolate (vaginitis)	a	yes
YJM975	Bergamo, Italy	Clinical isolate (vaginitis)	a	yes
FY4	Laboratory strain	Laboratory strain	a	no

Table S2 Qualitative scoring metrics

ASSAY	SCORE	CRITERIA
Complex colony morphology	0	simple colonies
	1	non-smooth colony surface
	2	signs of complex morphology
	3	moderate complex morphology (does not cover entire colony)
	4	strong complex morphology (covers entire colony)
	5	very strong complex morphology
Complex mat formation	0	simple mat
	1	very light sectoring and/or light ruffle on edge
	2	clear ruffle on edge or petals, can include sectoring
	3	obvious complexity at edges
	4	obvious complexity at edges and in center
	5	strong complexity across entirety of mat
Diploid filamentous growth	0	no filaments
	1	short filaments sparsely distributed around perimeter
	2	long filaments sparsely distributed around perimeter
	3	long filaments distributed around majority of perimeter
	4	moderate length filaments densely distributed around entire perimeter
	5	long filaments densely distributed around entire perimeter

File S1

Complete haploid phenotype dataset

File S1 is available for download as a .pdf at http://www.g3journal.org/lookup/suppl/doi:10.1534/g3.114.013250/-/DC1

Table S3 Qualitative score assignments

STRAIN	HAPLOID BIOREP1 COLONY	HAPLOID BIOREP2 COLONY	HAPLOID BIOREP1 MAT	DIPLOID COLONY	DIPLOID MAT
DBVPG6765	0	0	0	0	1
SK1	4	4	4	4	4
DBVPG6044	2	2	2	1	4
DBVPG1373	0	0	0	0	3
DBVPG1853	1	2	0	1	3
Y55	1	0	2	2	0
YPS128	3	3	4	2	0
DBVPG1106	0	0	0	0	1
DBVPG6040	4	3	4	1	2
BC187	2	2	5	0	1
YPS606	3	3	5	0	0
L-1374	3	3	4	2	2
L-1528	4	5	5	3	3
NCYC361	0	0	0	0	3
K11	3	3	5	3	4
Y12	0	0	0	0	1
YS2	0	0	1		
YS4	1	1	3		
YS9	0	0	2		
UWOPS83-787.3	1	1	0	0	0
UWOPS87-2421	1	1	0	1	0
UWOPS05-217.3	5	5	5	2	4
UWOPS05-227.2	5	5	5	1	4
W303	0	0	2		
322134S	5		5		
378604X	1	1	2		
273614N	1	1	0	0	1
YJM978	3	2	5	0	3
YJM981	5	5	5	3	2
YJM975	0	0	1	2	2
FY4	2	2	3		

Strains are assigned qualitative scores based on the metric described in Table S2.

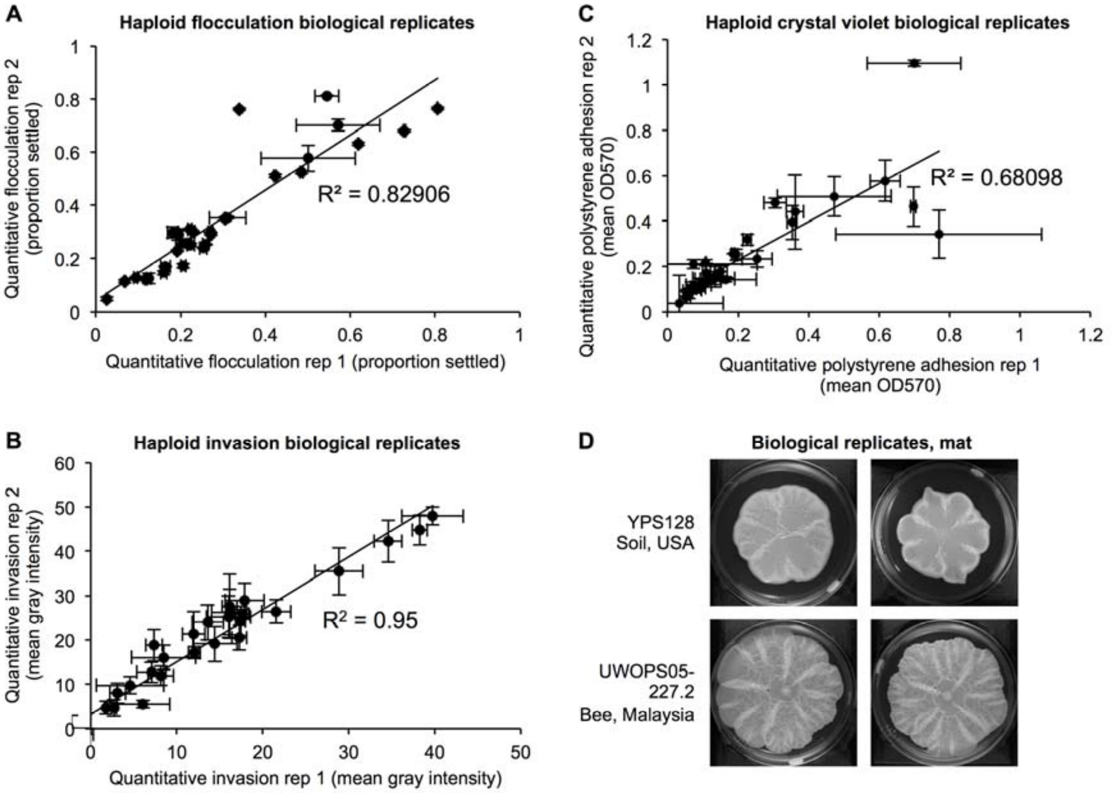

Figure S1 Haploid biological replicates demonstrate consistency of quantitative assays ***a*** Mean quantitative haploid flocculation data for one biological replicate (x-axis) is plotted against quantitative haploid flocculation data for a second biological replicate (y-axis) for correlation. Average taken and error bars calculated over three measurement replicates per strain. ***b*** Mean quantitative haploid invasion data for one biological replicate (x-axis) is plotted against quantitative haploid invasion data for a second biological replicate (y-axis) for correlation. Average taken and error bars calculated over three technical replicates per strain. ***c*** Mean quantitative haploid polystyrene adhesion data for one biological replicate (x-axis) is plotted against quantitative haploid polystyrene adhesion data for a second biological replicate (y-axis) for correlation. Average taken and error bars calculated over two technical replicates per strain. Outlier A shows the measurements from strain 322134S, and outlier B the measurements from strain SK1. Each of these strains was highly flocculent, and as a result the biofilm formed on polystyrene stained inconsistently across biological replicates and, in some cases, across technical replicates as well. If these outliers are eliminated from the analysis, the R^2 value becomes 0.83. ***d*** Biological replicates of complex mat formation for two biological replicates of representative haploid strains. Photos taken at day 13 of growth.

File S2

Complete prion-cured phenotype dataset

File S2 is available for download as a .pdf at http://www.g3journal.org/lookup/suppl/doi:10.1534/g3.114.013250/-/DC1

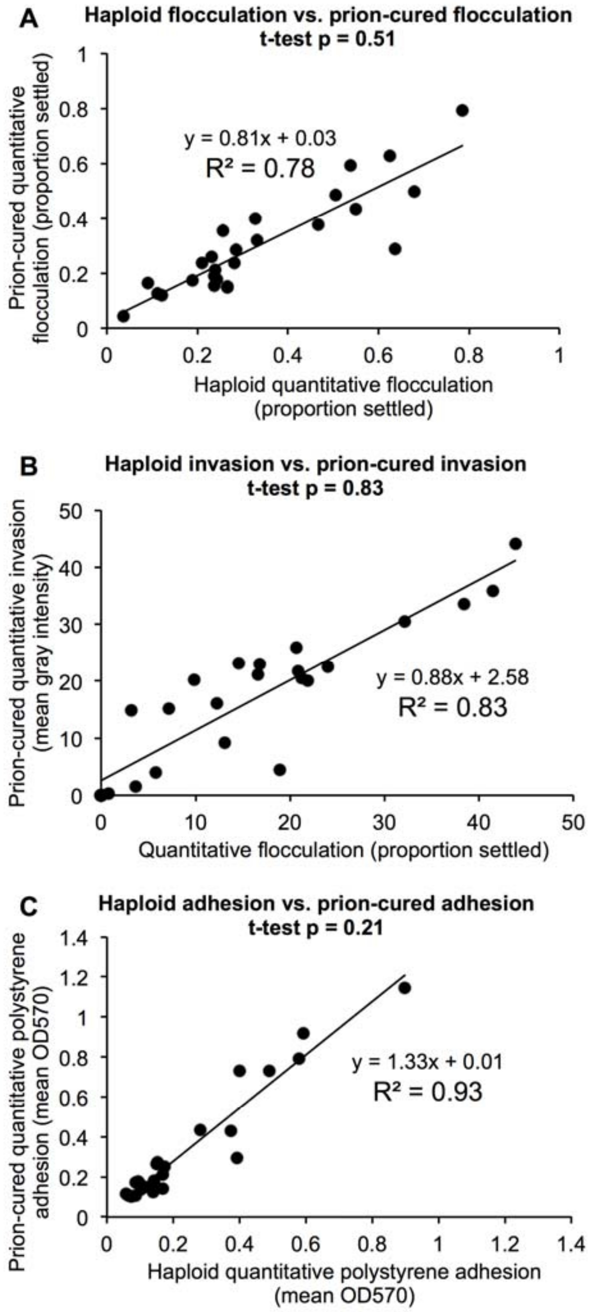

Figure S2 Prion-cured vs. haploid correlation plots show reproducibility ***a*** Mean quantitative haploid flocculation data for all biological replicates (x-axis) is plotted against quantitative prion-cured flocculation data across all cured replicates (y-axis) for correlation. P-value is shown for two-tailed t-test assuming unequal variance. ***b*** Mean quantitative haploid invasion data for all biological replicates (x-axis) is plotted against quantitative prion-cured invasion data across all cured replicates (y-axis) for correlation ***c*** Mean quantitative haploid polystyrene adhesion data for all biological replicates (x-axis) is plotted against quantitative prion-cured polystyrene adhesion data across all cured replicates (y-axis) for correlation

File S3

Table of quantitative assay results

File S3 is available for download as a tab-delimited .txt file at
http://www.g3journal.org/lookup/suppl/doi:10.1534/g3.114.013250/-/DC1

Table S4 Quantitative changes in prion-cured strains vs. haploid strains

	FLOCCULATION[a]	INVASION[b]	ADHESION[c]
Significant decrease by FDR correction*	5	1	0
No change by FDR correction	20	20	23
Significant increase by FDR correction	1	2	2

The numbers of prion-cured strains that showed differences when compared to the original haploid strains across three quantitative metrics are shown. Quantitative values were compared using a two-tailed t-test assuming unequal variance across all technical replicates for each strain. *P*-values were evaluated for significance using the *q*-values package from Storey with the Benjamini-Hochberg method (Storey 2002).

* FDR = 0.05
[a] 26 strains
[b] 23 strains
[c] 25 strains

DBVPG6765 (YMD1152)

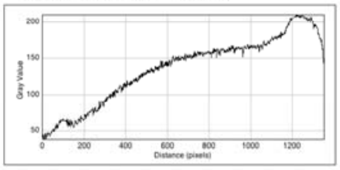

DBVPG1106 (YMD1166)

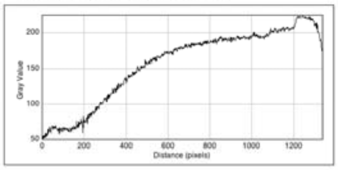

K11 (YMD1180)

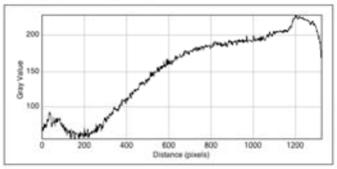

SK1 (YMD1154)

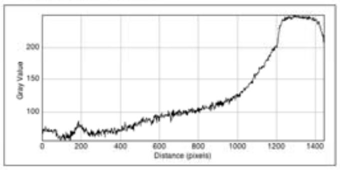

DBVPG6040 (YMD1168)

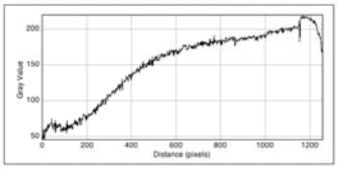

Y12 (YMD1182)

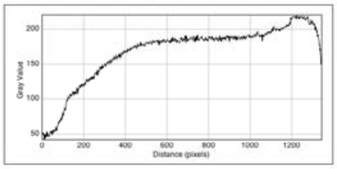

DBVPG6044 (YMD1156)

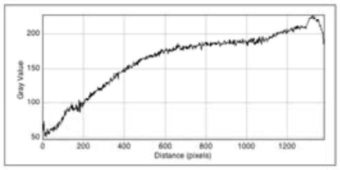

BC187 (YMD1170)

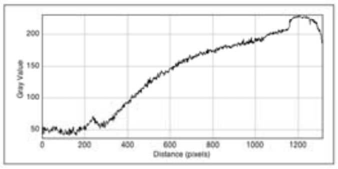

YS2 (YMD1184)

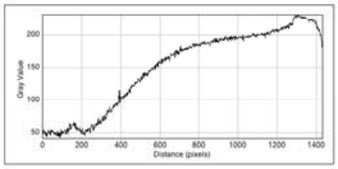

DBVPG1373 (YMD1158)

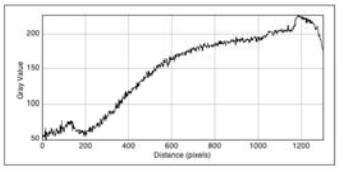

YPS606 (YMD1172)

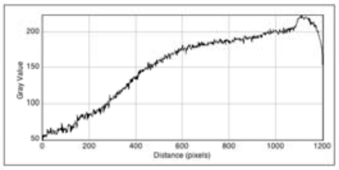

YS4 (YMD1186)

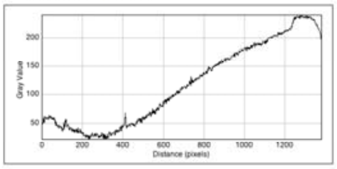

DBVPG1853 (YMD1160)

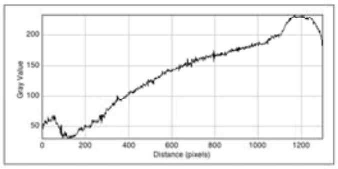

L-1374 (YMD1174)

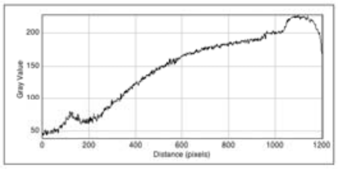

YS9 (YMD1188)

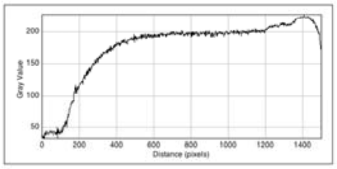

Y55 (YMD1162)

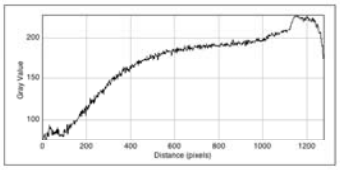

L-1528 (YMD1176)

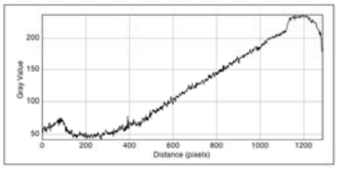

UWOPS83-787.3 (YMD1190)

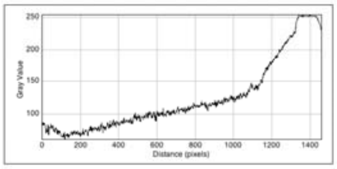

YPS128 (YMD1164)

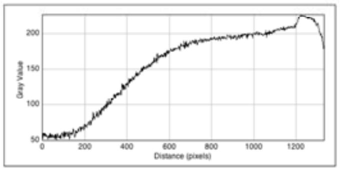

NCYC361 (YMD1178)

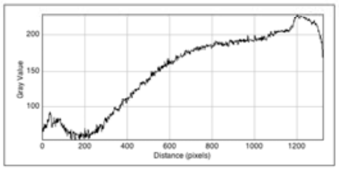

UWOPS87-242.1 (YMD1192)

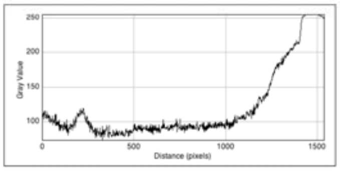

UWOPS05-217.3 (YMD1194)

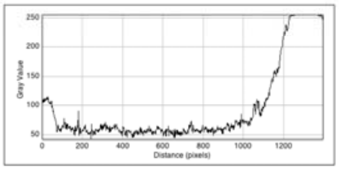

378604X (YMD1202)

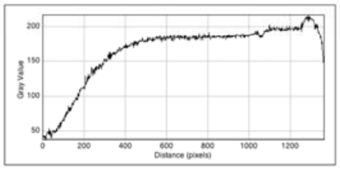

YJM975 (YMD1210)

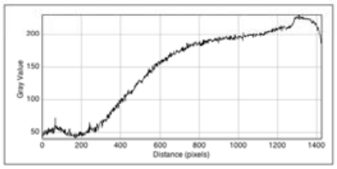

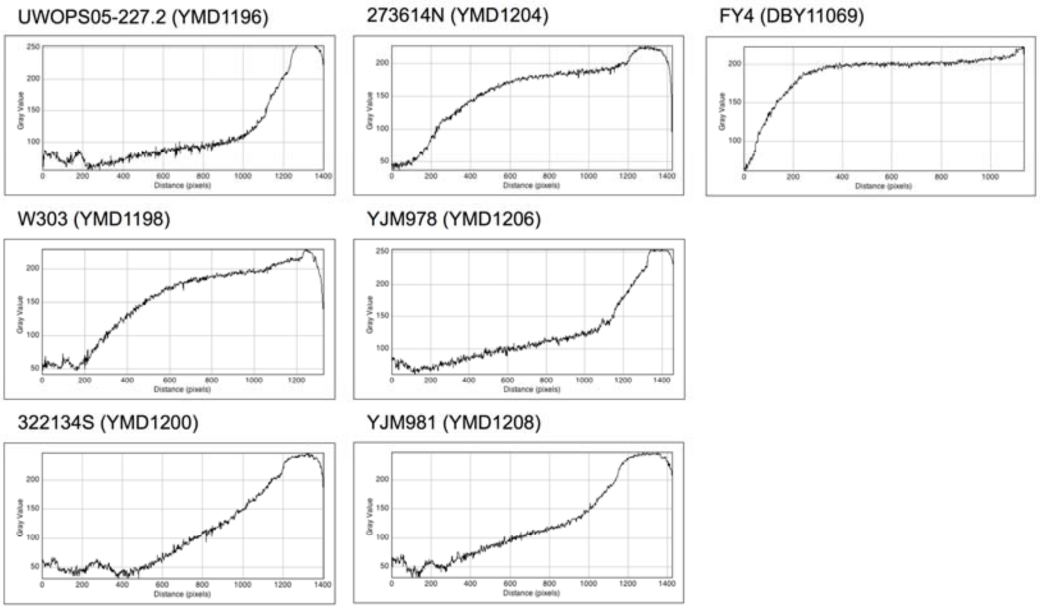

Figure S3 Haploid settling plot profiles A representative plot profile generated by ImageJ is shown for each strain in one biological replicate of the haploid quantitative flocculation assay. Plot profiles show mean gray value along a line drawn on the 60-minute time point settling image from the meniscus of the culture to the bottom of the tube with corresponding pixel distance values on the x-axis of the plot.

DBVPG6765 (YMD2348)

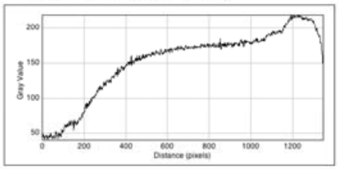

DBVPG1106 (YMD2355)

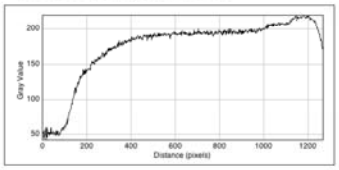

K11 (YMD2362)

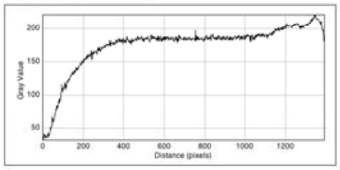

SK1 (YMD2349)

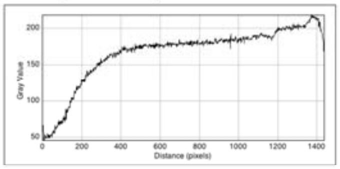

DBVPG6040 (YMD2356)

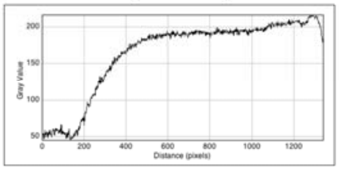

Y12 (YMD2363)

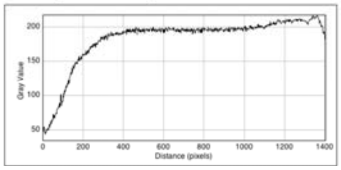

DBVPG6044 (YMD2350)

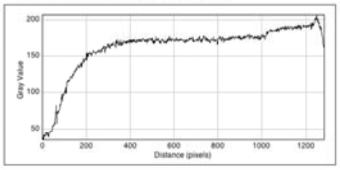

BC187 (YMD2357)

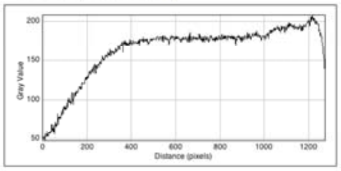

YS2 (YMD2364)

Diploid not tested

DBVPG1373 (YMD2351)

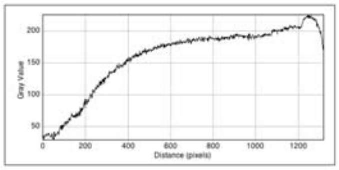

YPS606 (YMD2358)

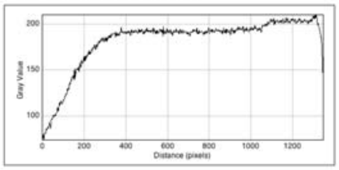

YS4 (YMD2365)

Diploid not tested

DBVPG1853 (YMD2352)

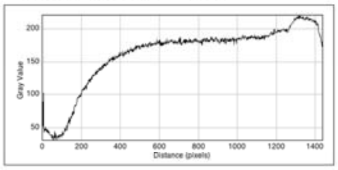

L-1374 (YMD2359)

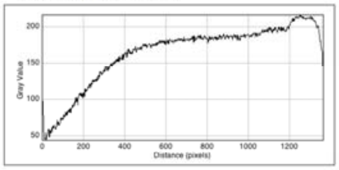

YS9 (YMD2366)

Diploid not tested

Y55 (YMD2353)

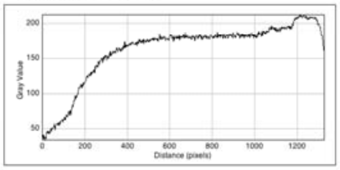

L-1528 (YMD2360)

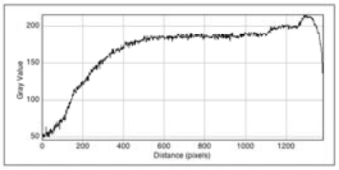

UWOPS83-787.3 (YMD2367)

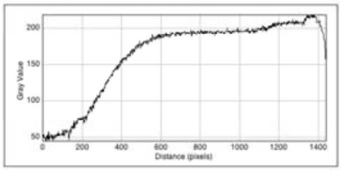

YPS128 (YMD2354)

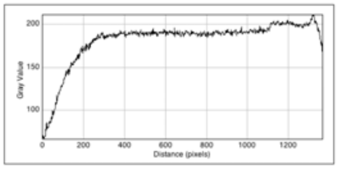

NCYC361 (YMD2361)

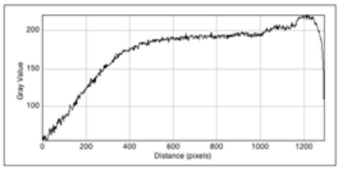

UWOPS87-242.1 (YMD2368)

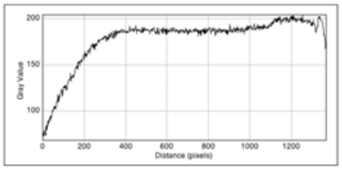

UWOPS05-217.3 (YMD2369)

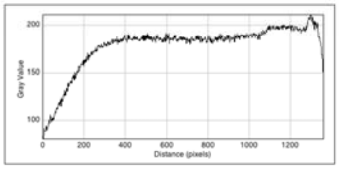

378604X (YMD2373)

Diploid not tested

YJM975 (YMD2377)

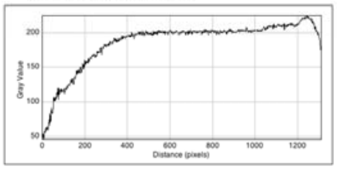

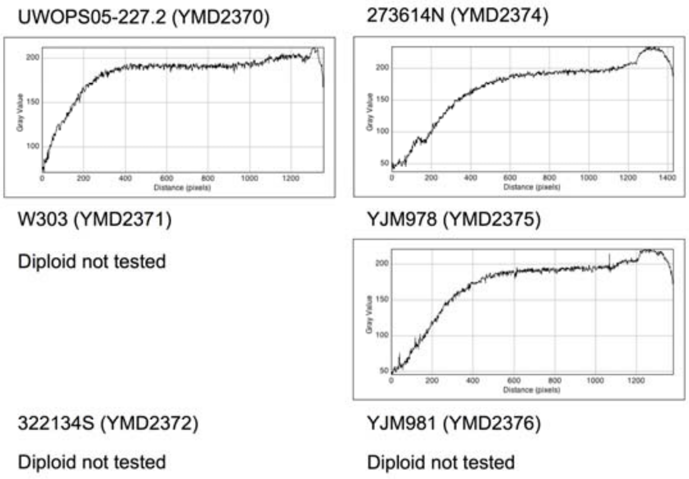

Figure S4 Diploid settling plot profiles A representative plot profile generated by ImageJ is shown for each strain in the diploid quantitative flocculation assay. Plot profiles show mean gray value along a line drawn on the 60-minute time point settling image from the meniscus of the culture to the bottom of the tube with corresponding pixel distance values on the x-axis of the plot.

File S4

Complete diploid phenotype dataset

File S4 is available for download as a .pdf at http://www.g3journal.org/lookup/suppl/doi:10.1534/g3.114.013250/-/DC1
