# Supplementary material for: Ploidy-Regulated Variation in Biofilm-Related Phenotypes in Natural Isolates of Saccharomyces cerevisiae
Source: G3 (Bethesda). 2014 Jul 24;4(9):1773–86. doi: 10.1534/g3.114.013250 (PMC4169170; doi:10.1534/g3.114.013250)
Supplement: Supporting Information [file supp_g3.114.013250_FigureS1.pdf]

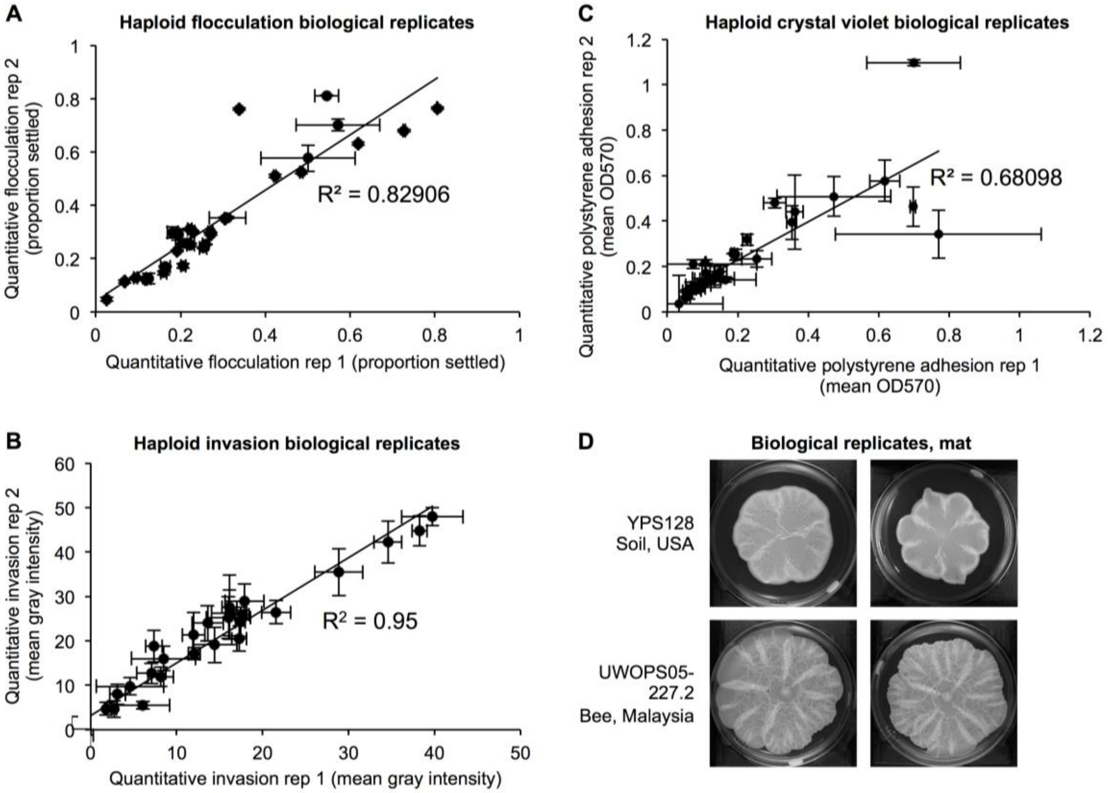

Figure S1 Haploid biological replicates demonstrate consistency of quantitative assays ***a*** Mean quantitative haploid flocculation data for one biological replicate (x-axis) is plotted against quantitative haploid flocculation data for a second biological replicate (y-axis) for correlation. Average taken and error bars calculated over three measurement replicates per strain. ***b*** Mean quantitative haploid invasion data for one biological replicate (x-axis) is plotted against quantitative haploid invasion data for a second biological replicate (y-axis) for correlation. Average taken and error bars calculated over three technical replicates per strain. ***c*** Mean quantitative haploid polystyrene adhesion data for one biological replicate (x-axis) is plotted against quantitative haploid polystyrene adhesion data for a second biological replicate (y-axis) for correlation. Average taken and error bars calculated over two technical replicates per strain. Outlier A shows the measurements from strain 322134S, and outlier B the measurements from strain SK1. Each of these strains was highly flocculent, and as a result the biofilm formed on polystyrene stained inconsistently across biological replicates and, in some cases, across technical replicates as well. If these outliers are eliminated from the analysis, the R^2 value becomes 0.83. ***d*** Biological replicates of complex mat formation for two biological replicates of representative haploid strains. Photos taken at day 13 of growth.
