# Supplementary material for: Ploidy-Regulated Variation in Biofilm-Related Phenotypes in Natural Isolates of Saccharomyces cerevisiae
Source: G3 (Bethesda). 2014 Jul 24;4(9):1773–86. doi: 10.1534/g3.114.013250 (PMC4169170; doi:10.1534/g3.114.013250)
Supplement: Supporting Information [file supp_g3.114.013250_FigureS2.pdf]

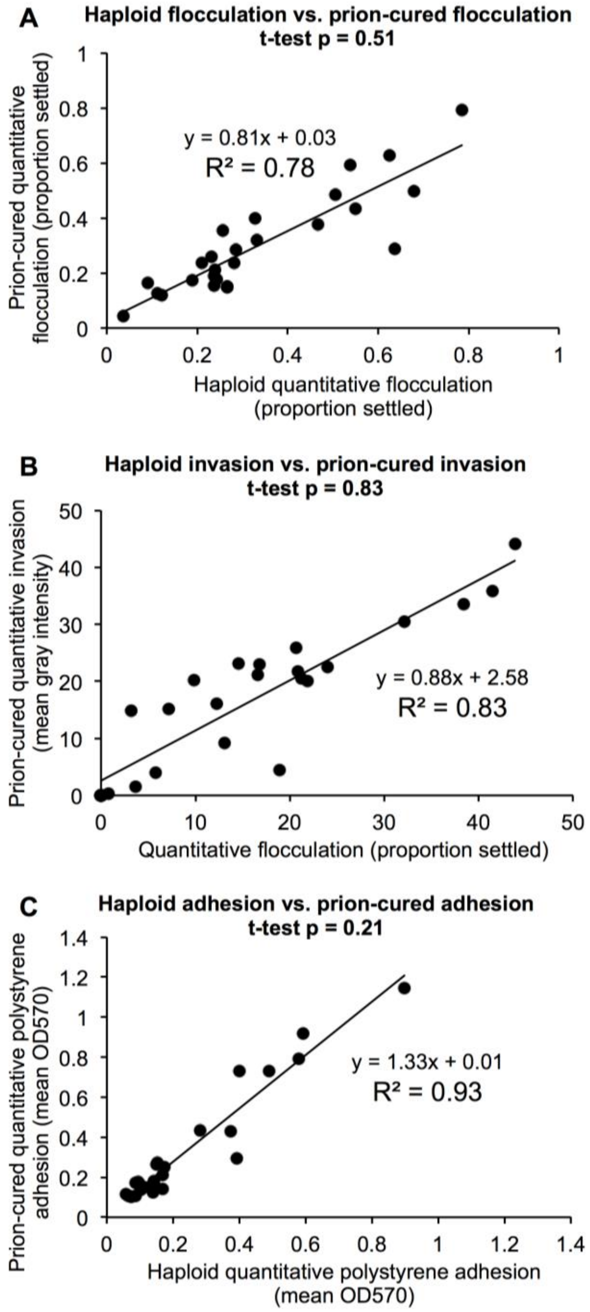

Figure S2 Prion-cured vs. haploid correlation plots show reproducibility ***a*** Mean quantitative haploid flocculation data for all biological replicates (x-axis) is plotted against quantitative prion-cured flocculation data across all cured replicates (y-axis) for correlation. P-value is shown for two-tailed t-test assuming unequal variance. ***b*** Mean quantitative haploid invasion data for all biological replicates (x-axis) is plotted against quantitative prion-cured invasion data across all cured replicates (y-axis) for correlation ***c*** Mean quantitative haploid polystyrene adhesion data for all biological replicates (x-axis) is plotted against quantitative prion-cured polystyrene adhesion data across all cured replicates (y-axis) for correlation
