# Supplementary material for: Ploidy-Regulated Variation in Biofilm-Related Phenotypes in Natural Isolates of Saccharomyces cerevisiae
Source: G3 (Bethesda). 2014 Jul 24;4(9):1773–86. doi: 10.1534/g3.114.013250 (PMC4169170; doi:10.1534/g3.114.013250)
Supplement: Supporting Information [file supp_g3.114.013250_FigureS3.pdf]

DBVPG6765 (YMD1152)

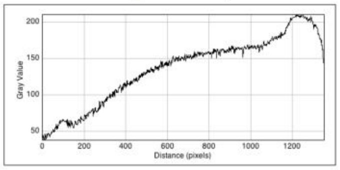

DBVPG1106 (YMD1166)

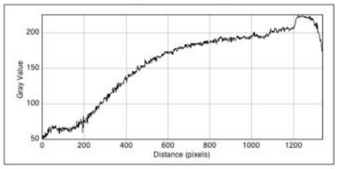

K11 (YMD1180)

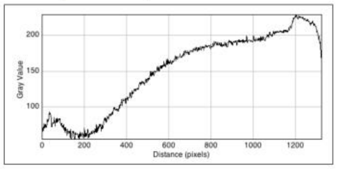

SK1 (YMD1154)

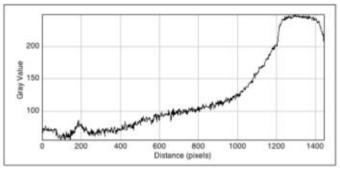

DBVPG6040 (YMD1168)

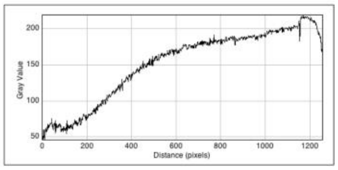

Y12 (YMD1182)

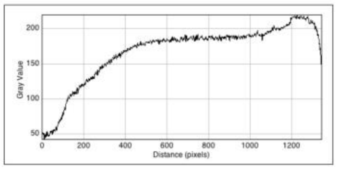

DBVPG6044 (YMD1156)

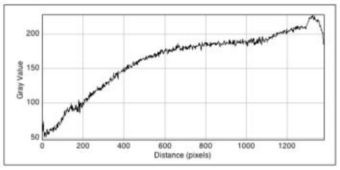

BC187 (YMD1170)

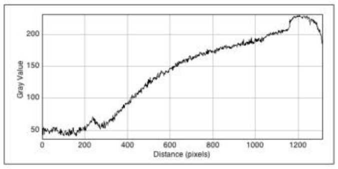

YS2 (YMD1184)

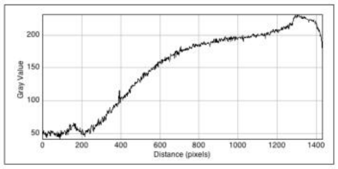

DBVPG1373 (YMD1158)

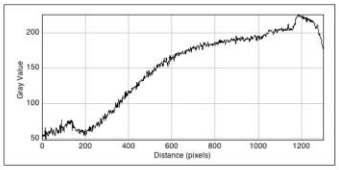

YPS606 (YMD1172)

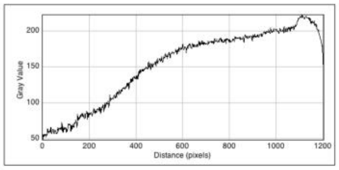

YS4 (YMD1186)

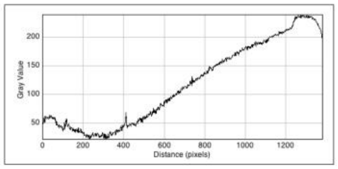

DBVPG1853 (YMD1160)

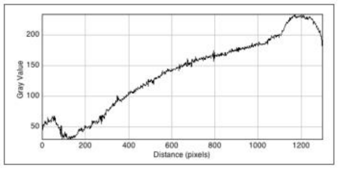

L-1374 (YMD1174)

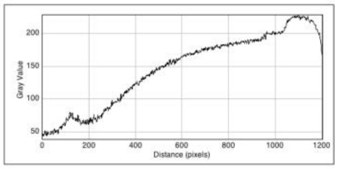

YS9 (YMD1188)

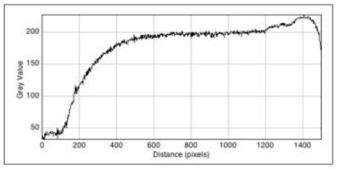

Y55 (YMD1162)

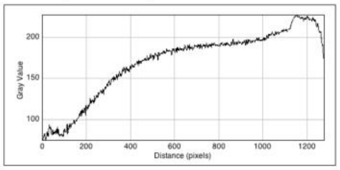

L-1528 (YMD1176)

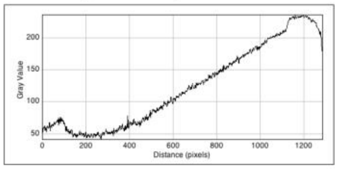

UWOPS83-787.3 (YMD1190)

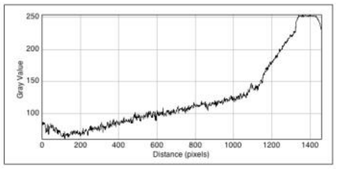

YPS128 (YMD1164)

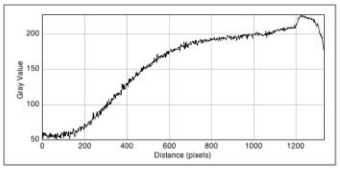

NCYC361 (YMD1178)

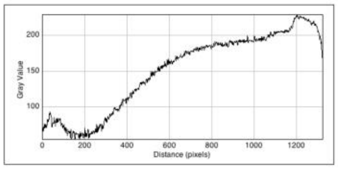

UWOPS87-242.1 (YMD1192)

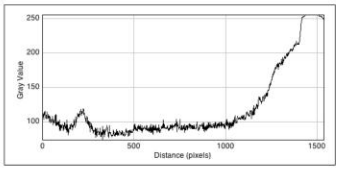

UWOPS05-217.3 (YMD1194)

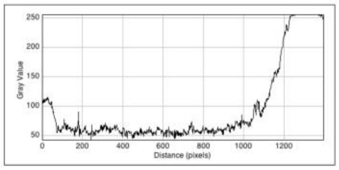

378604X (YMD1202)

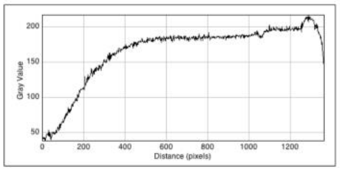

YJM975 (YMD1210)

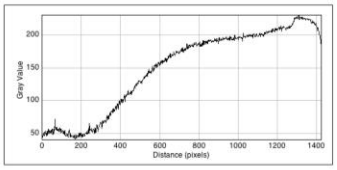

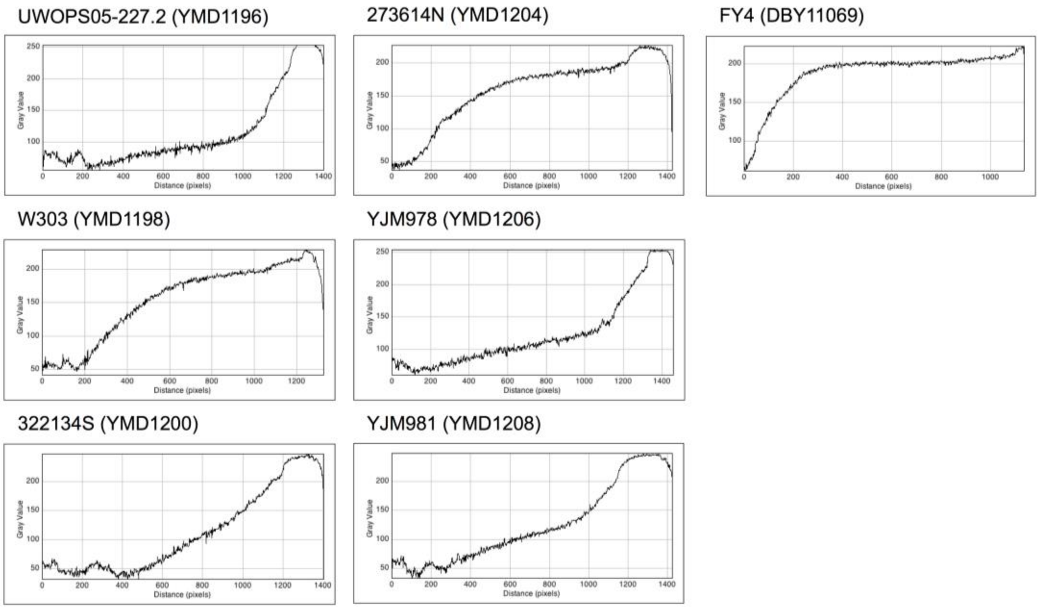

Figure S3 Haploid settling plot profiles A representative plot profile generated by ImageJ is shown for each strain in one biological replicate of the haploid quantitative flocculation assay. Plot profiles show mean gray value along a line drawn on the 60-minute time point settling image from the meniscus of the culture to the bottom of the tube with corresponding pixel distance values on the x-axis of the plot.
