# Supplementary material for: Ploidy-Regulated Variation in Biofilm-Related Phenotypes in Natural Isolates of Saccharomyces cerevisiae
Source: G3 (Bethesda). 2014 Jul 24;4(9):1773–86. doi: 10.1534/g3.114.013250 (PMC4169170; doi:10.1534/g3.114.013250)
Supplement: Supporting Information [file supp_g3.114.013250_FigureS4.pdf]

DBVPG6765 (YMD2348)

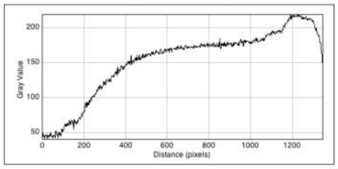

DBVPG1106 (YMD2355)

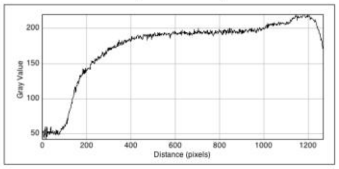

K11 (YMD2362)

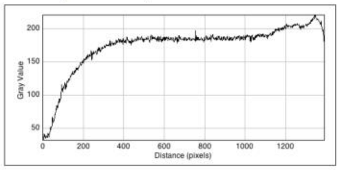

SK1 (YMD2349)

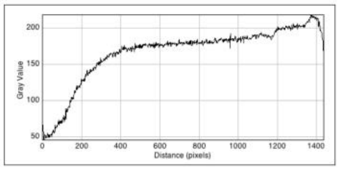

DBVPG6040 (YMD2356)

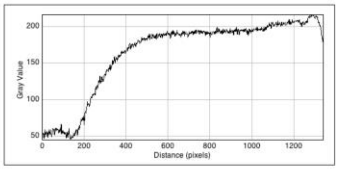

Y12 (YMD2363)

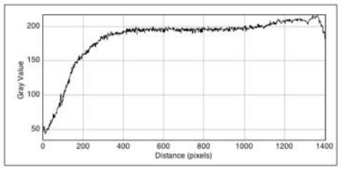

DBVPG6044 (YMD2350)

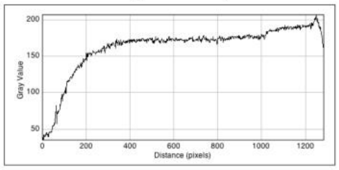

BC187 (YMD2357)

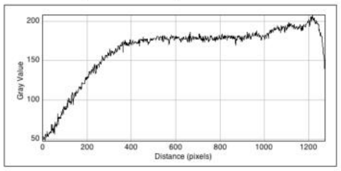

YS2 (YMD2364)

Diploid not tested

DBVPG1373 (YMD2351)

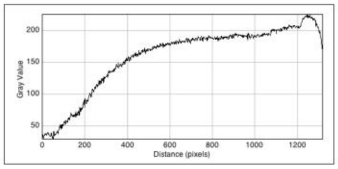

YPS606 (YMD2358)

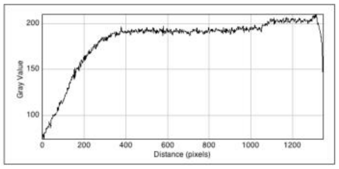

YS4 (YMD2365)

Diploid not tested

DBVPG1853 (YMD2352)

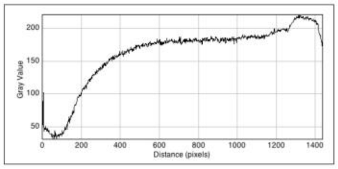

L-1374 (YMD2359)

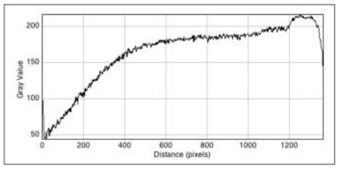

YS9 (YMD2366)

Diploid not tested

Y55 (YMD2353)

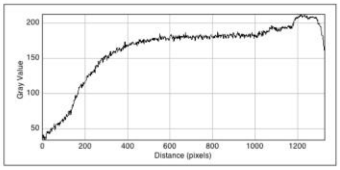

L-1528 (YMD2360)

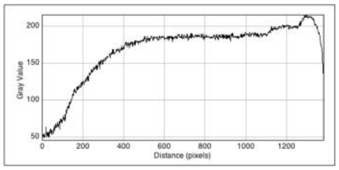

UWOPS83-787.3 (YMD2367)

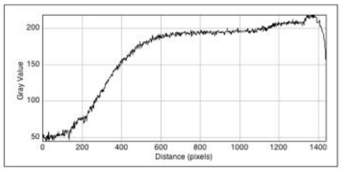

YPS128 (YMD2354)

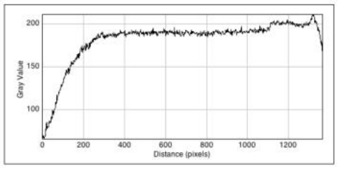

NCYC361 (YMD2361)

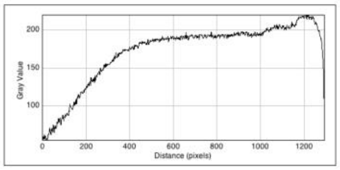

UWOPS87-242.1 (YMD2368)

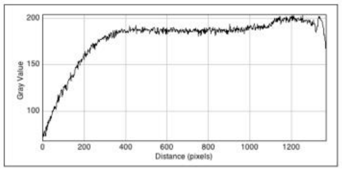

UWOPS05-217.3 (YMD2369)

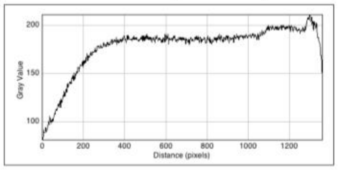

378604X (YMD2373)

Diploid not tested

YJM975 (YMD2377)

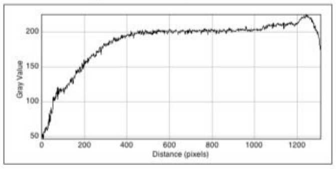

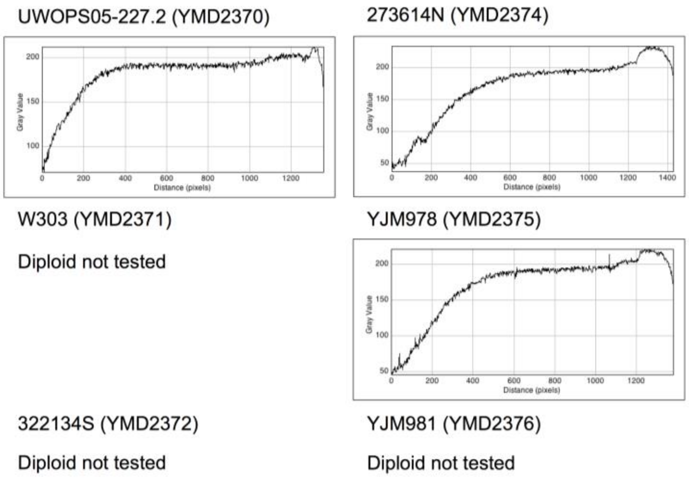

Figure S4 Diploid settling plot profiles A representative plot profile generated by ImageJ is shown for each strain in the diploid quantitative flocculation assay. Plot profiles show mean gray value along a line drawn on the 60-minute time point settling image from the meniscus of the culture to the bottom of the tube with corresponding pixel distance values on the x-axis of the plot.
