# Supplementary material for: Ploidy-Regulated Variation in Biofilm-Related Phenotypes in Natural Isolates of Saccharomyces cerevisiae
Source: G3 (Bethesda). 2014 Jul 24;4(9):1773–86. doi: 10.1534/g3.114.013250 (PMC4169170; doi:10.1534/g3.114.013250)
Supplement: Supporting Information [file supp_g3.114.013250_FileS1.pdf]

File S1A: Haploid phenotype panel, biological replicate 1

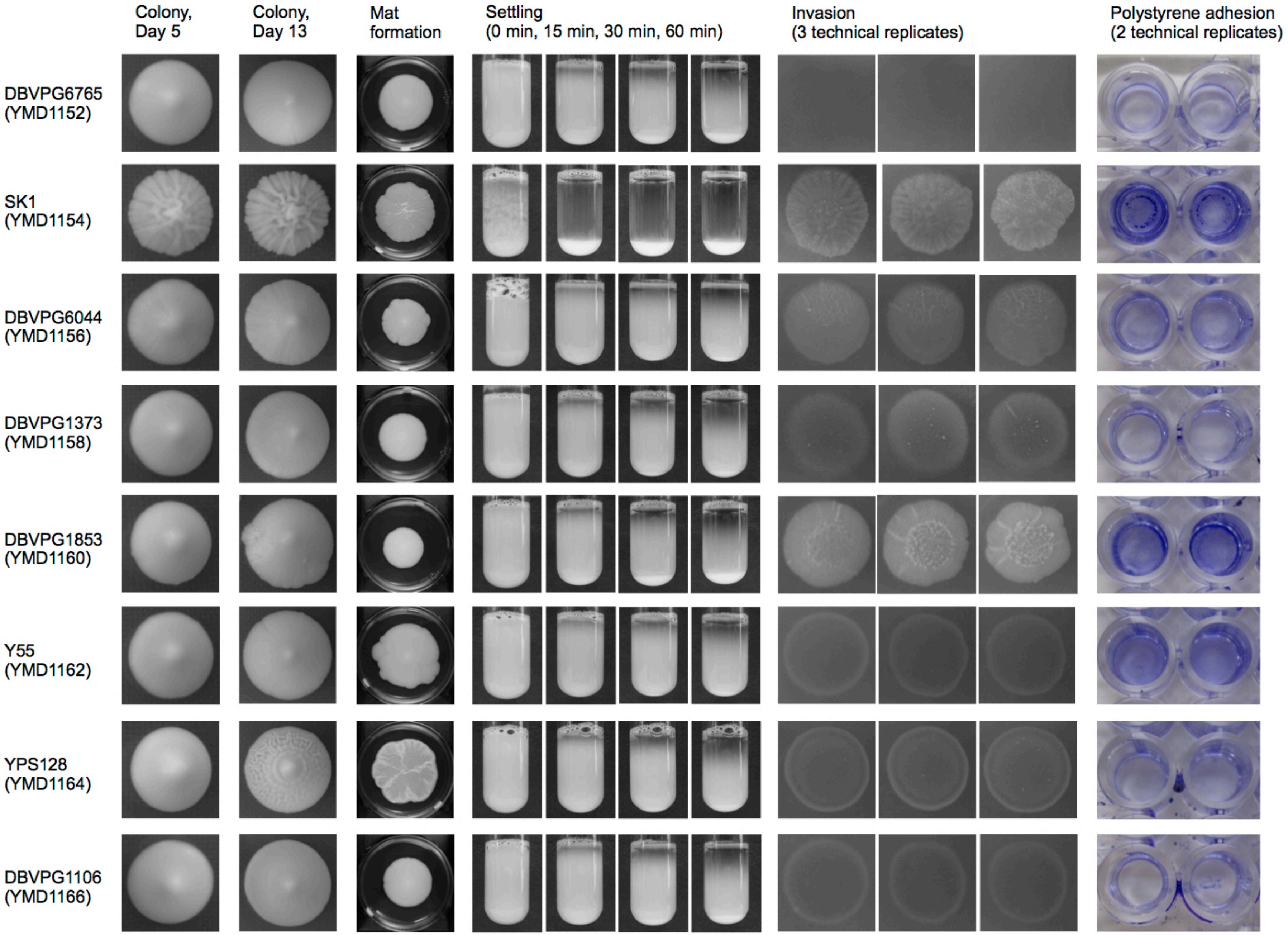

File S1A: Haploid phenotype panel, biological replicate 1

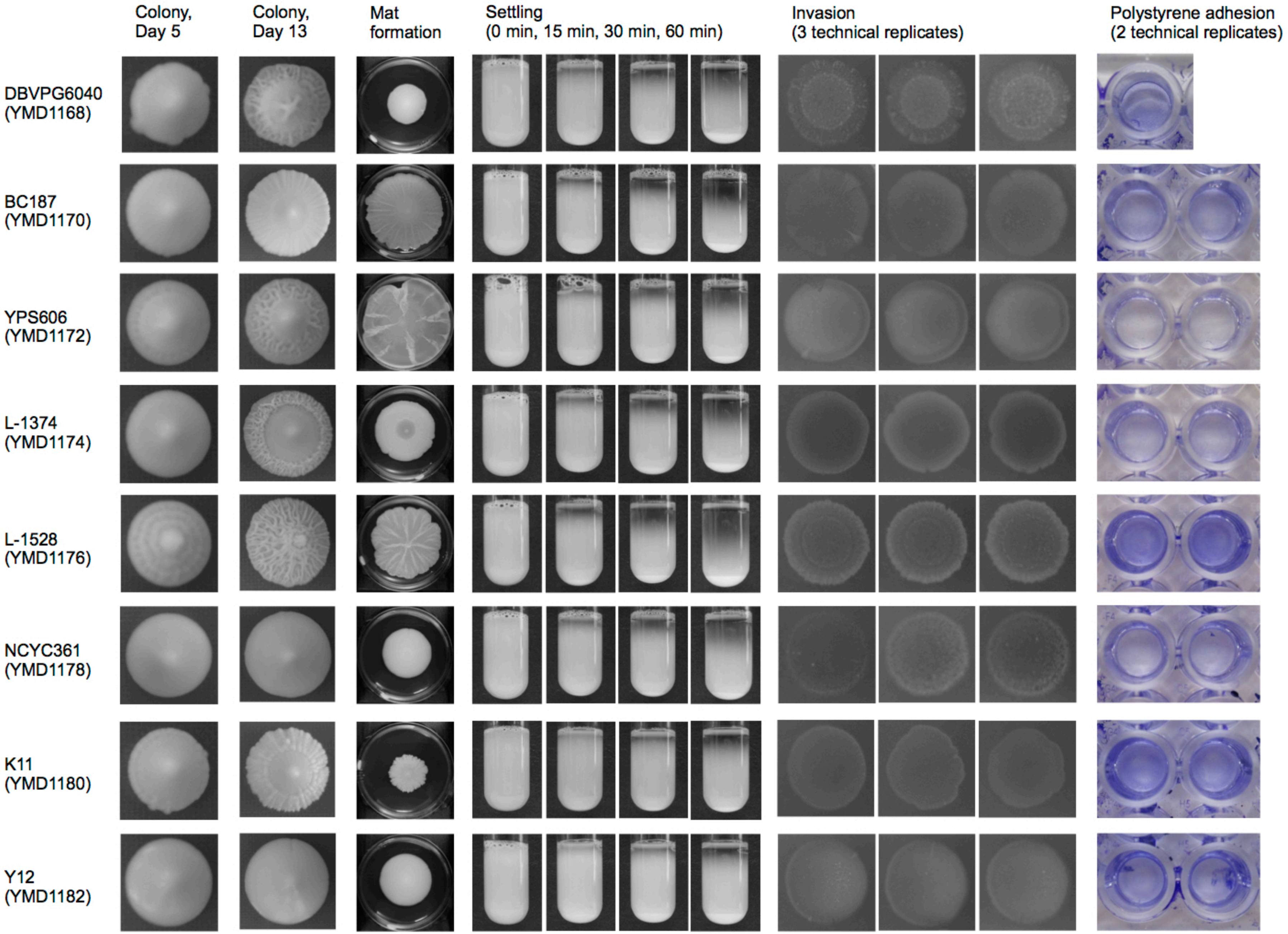

File S1A: Haploid phenotype panel, biological replicate 1

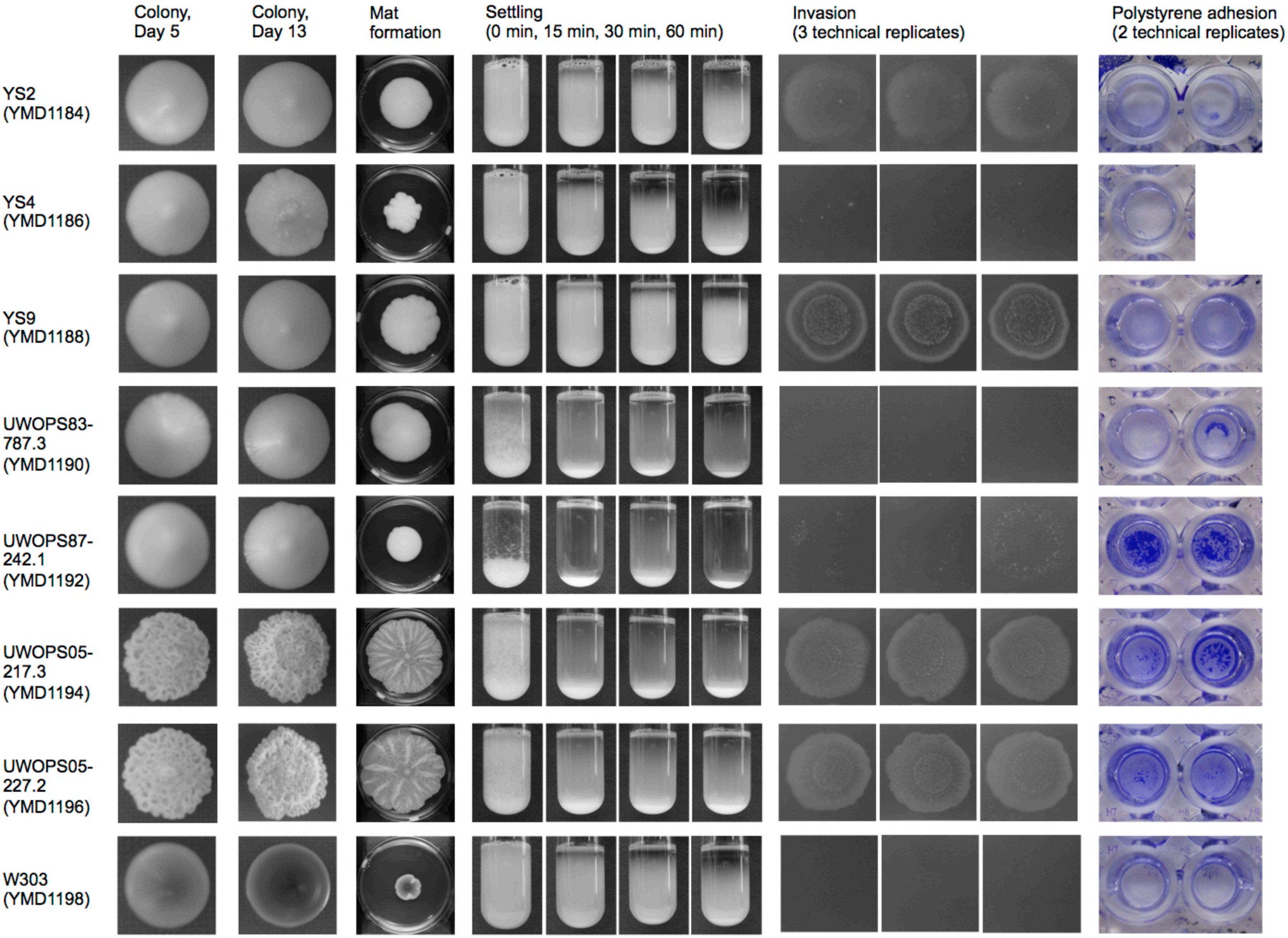

File S1A: Haploid phenotype panel, biological replicate 1

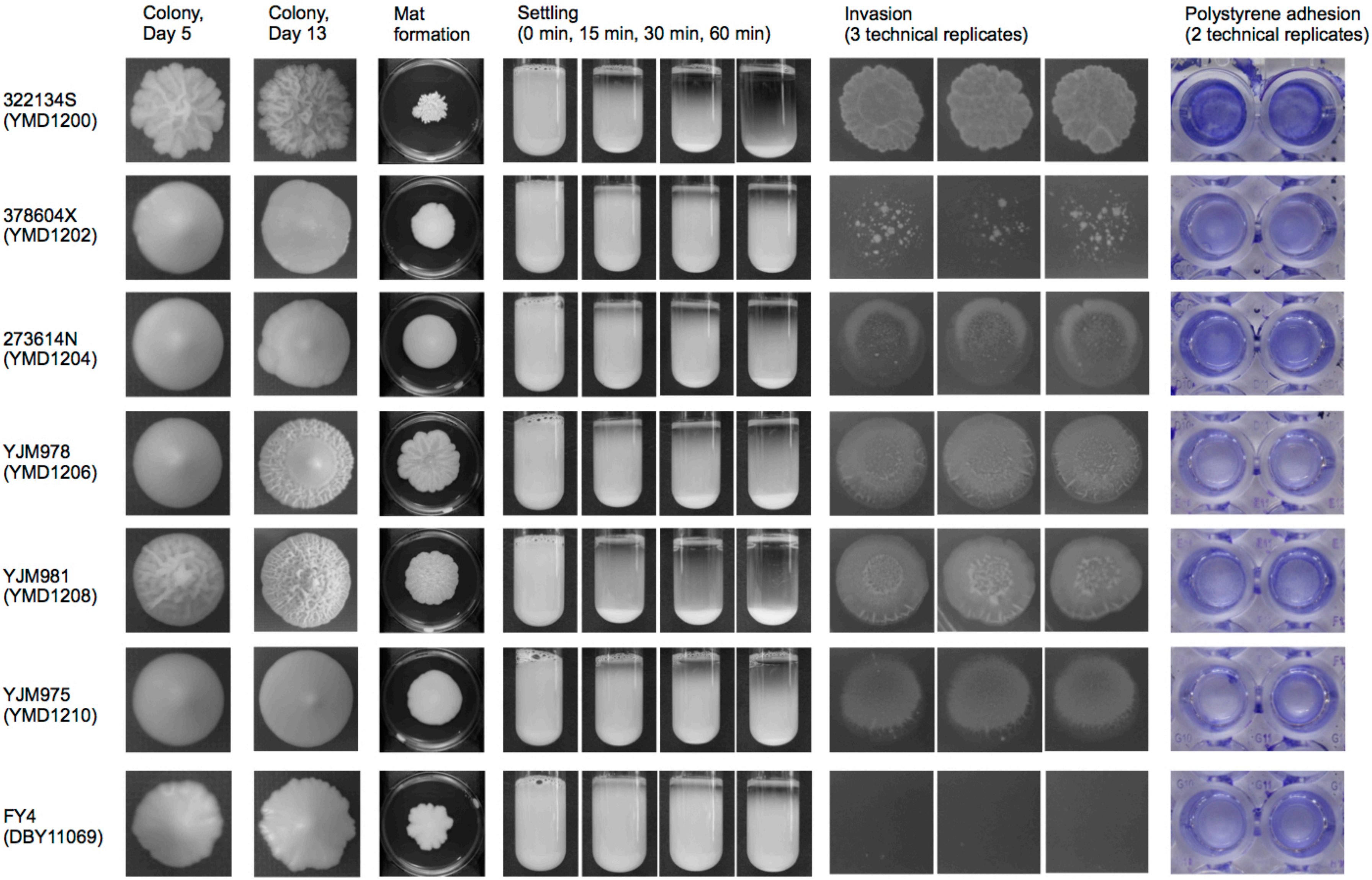

File S1B: Haploid phenotype panel, biological replicate 2

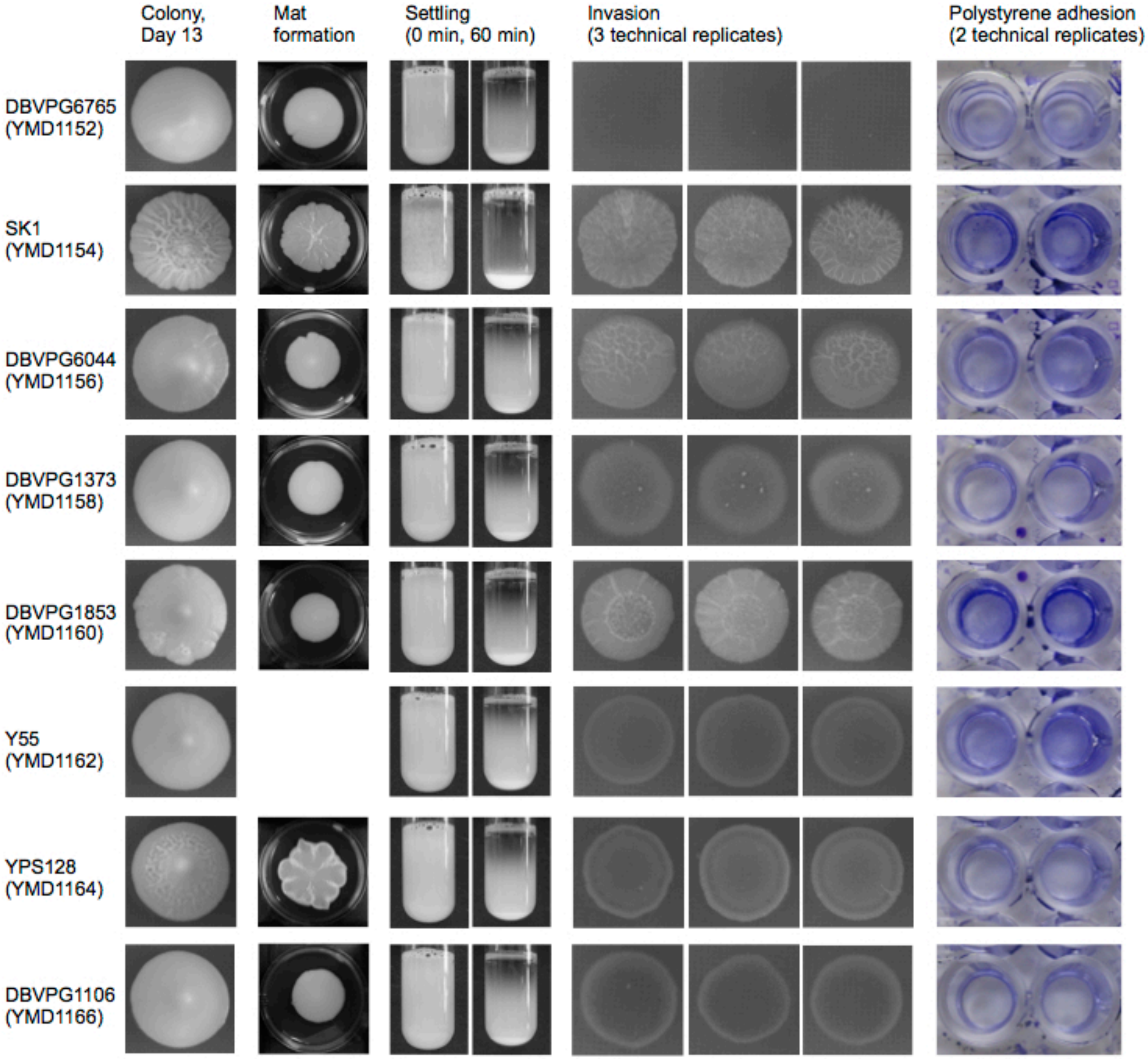

File S1B: Haploid phenotype panel, biological replicate 2

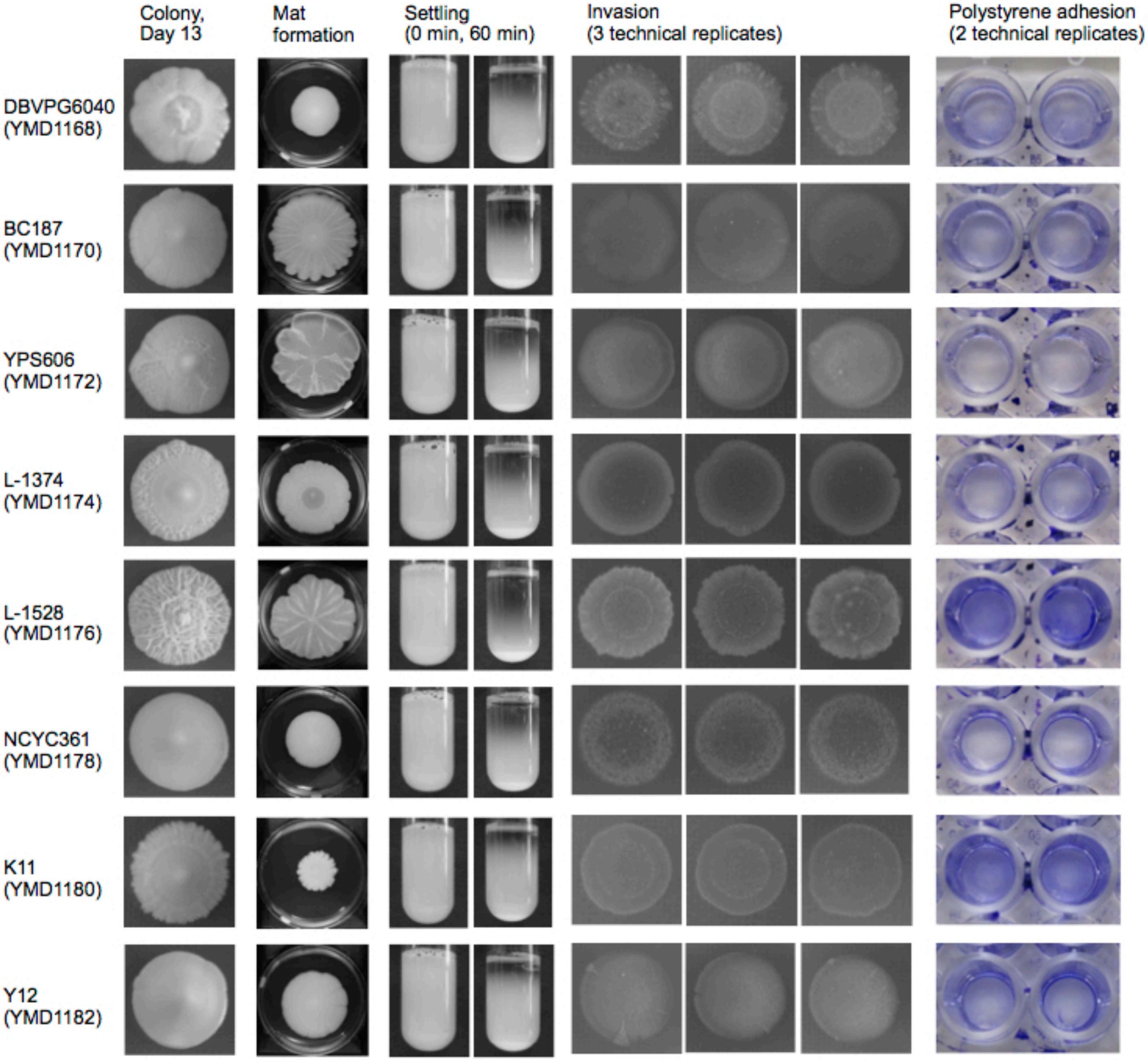

File S1B: Haploid phenotype panel, biological replicate 2

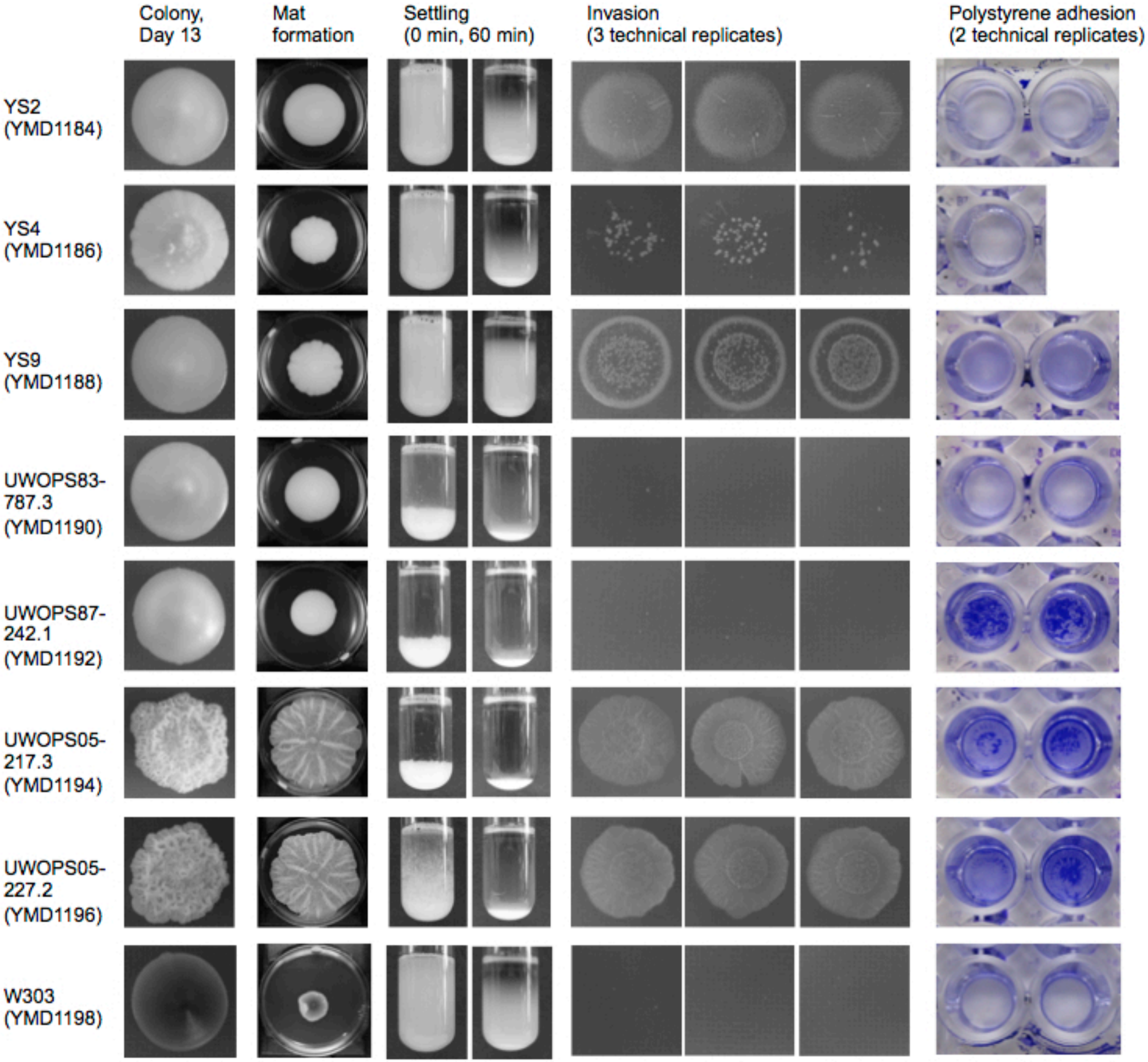

File S1B: Haploid phenotype panel, biological replicate 2

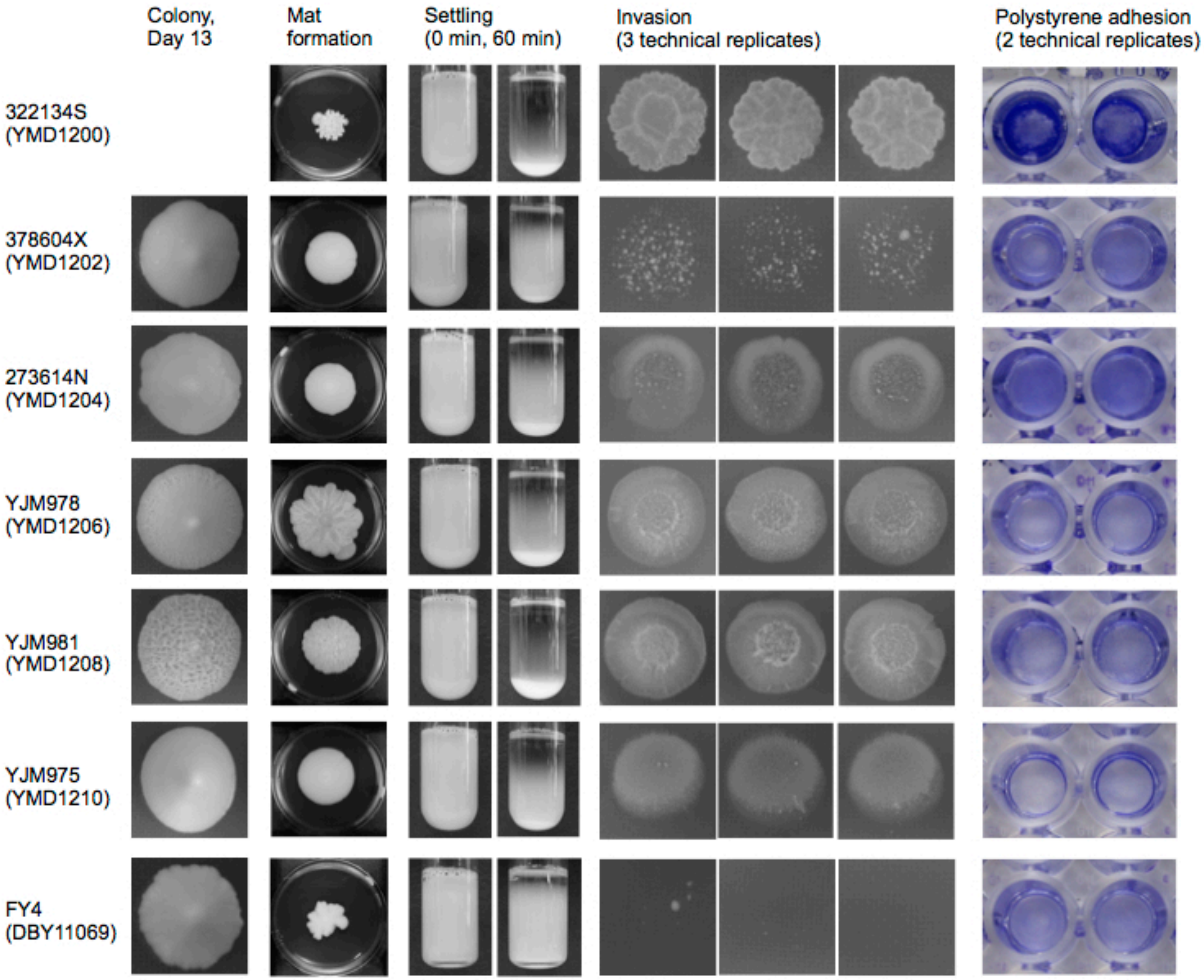

File S1 Complete haploid phenotypic panel Full phenotypic panel for all 31 haploid strains used in study. Strains are listed with their formal name and origin and are shown across five different phenotypes. Complex mat formation images include the plate for scale. Three technical replicates are shown for the invasion assay, photographed after 24 hours' growth following washing on day 5. Two technical replicates are shown for the polystyrene adhesion assay. Pictured biofilms are fixed and stained with a 1% w/v crystal violet solution. Parts ***a*** and ***b*** show data from separate haploid biological replicates.
