# Supplementary material for: Ploidy-Regulated Variation in Biofilm-Related Phenotypes in Natural Isolates of Saccharomyces cerevisiae
Source: G3 (Bethesda). 2014 Jul 24;4(9):1773–86. doi: 10.1534/g3.114.013250 (PMC4169170; doi:10.1534/g3.114.013250)
Supplement: Supporting Information [file supp_g3.114.013250_FileS2.pdf]

File S2: Prion-cured phenotype panel

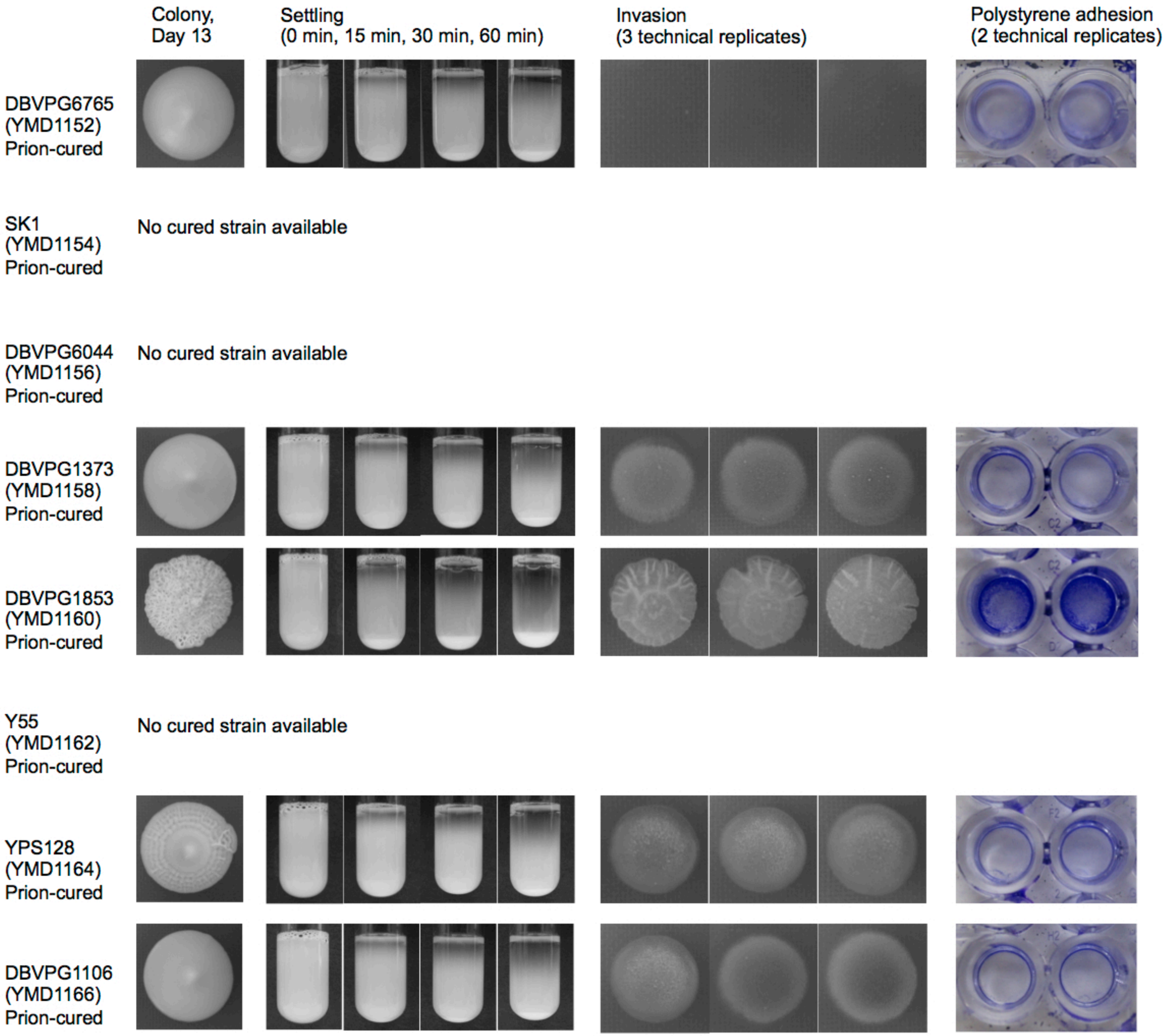

File S2: Prion-cured phenotype panel

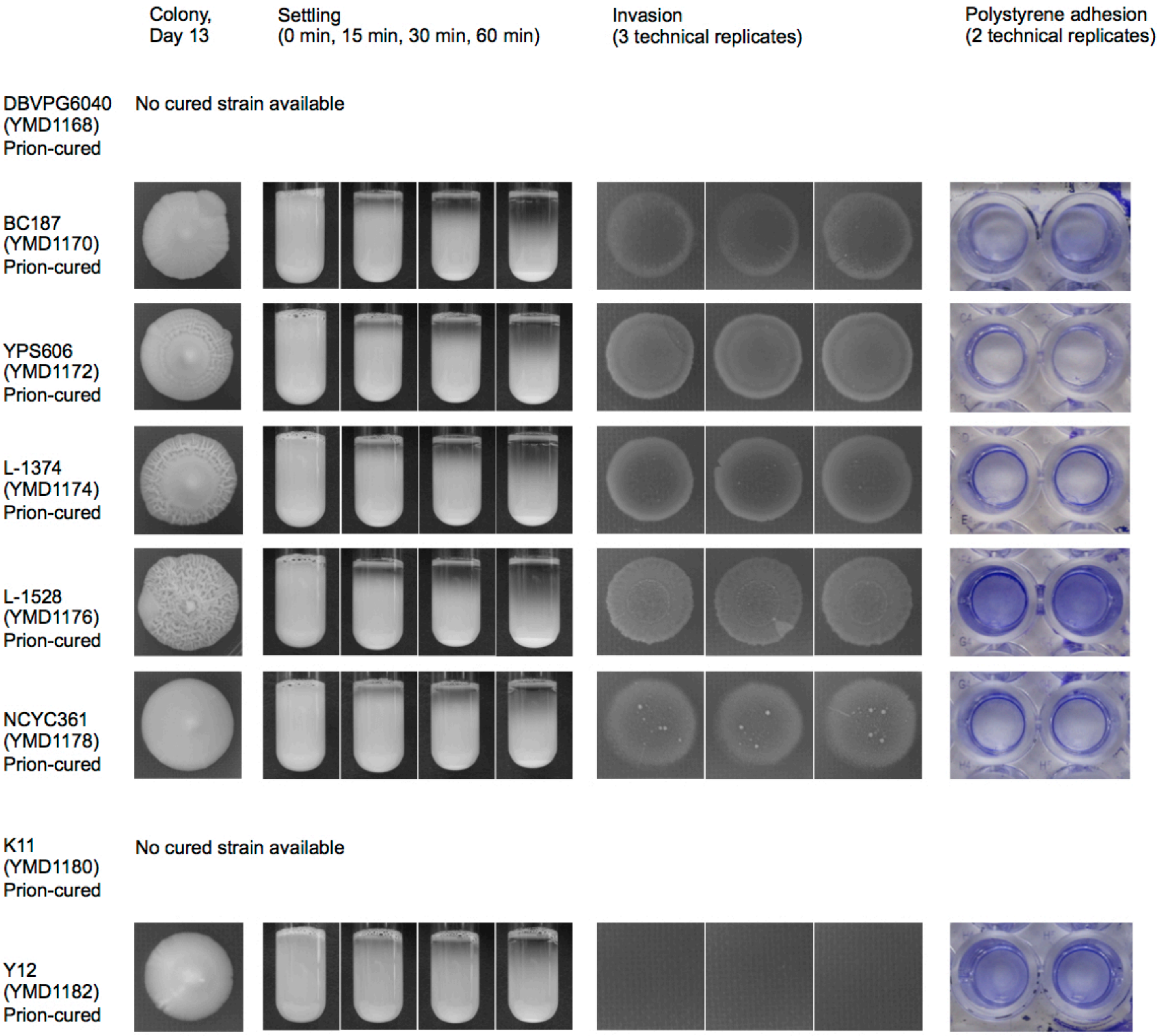

File S2: Prion-cured phenotype panel

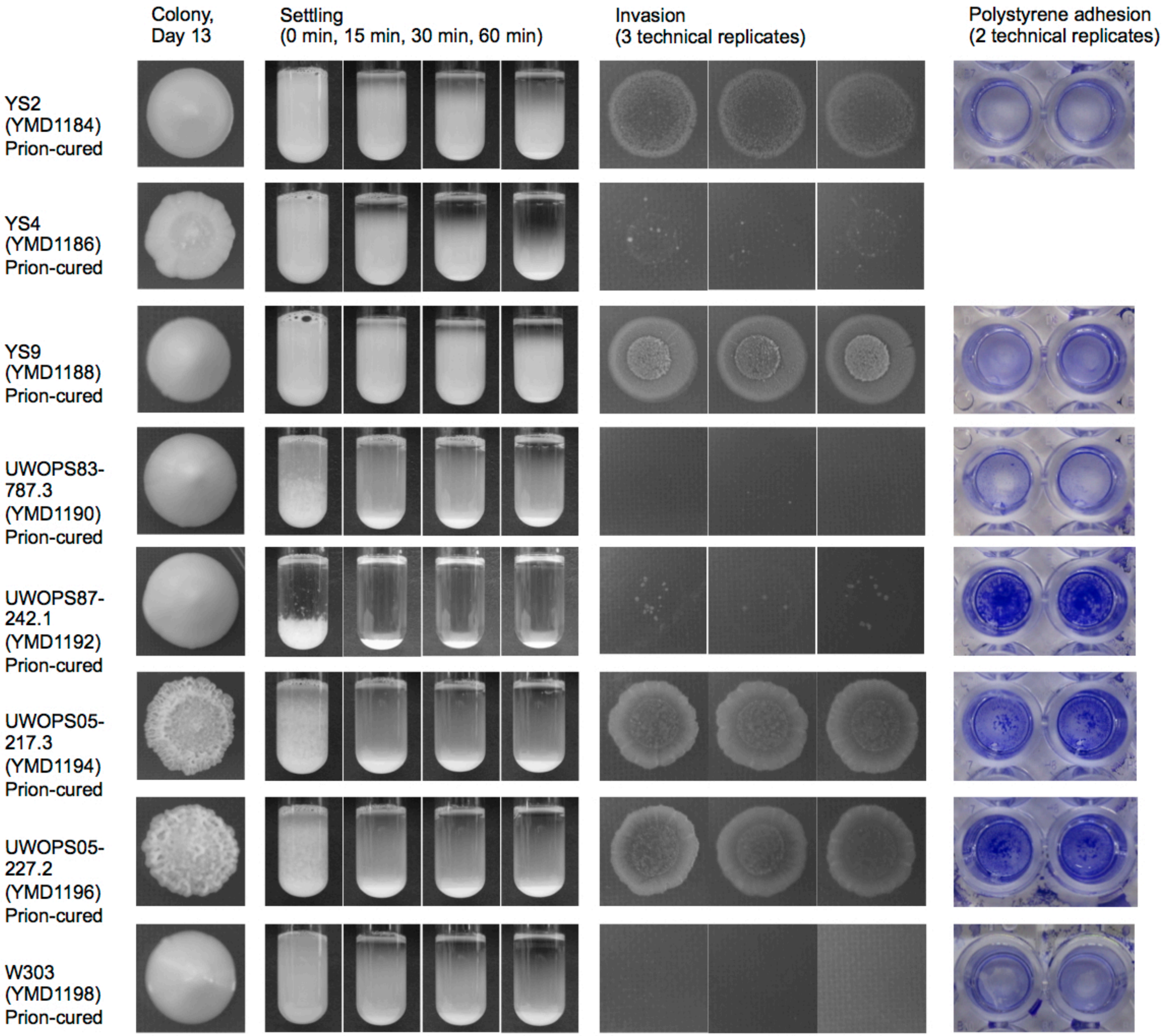

File S2: Prion-cured phenotype panel

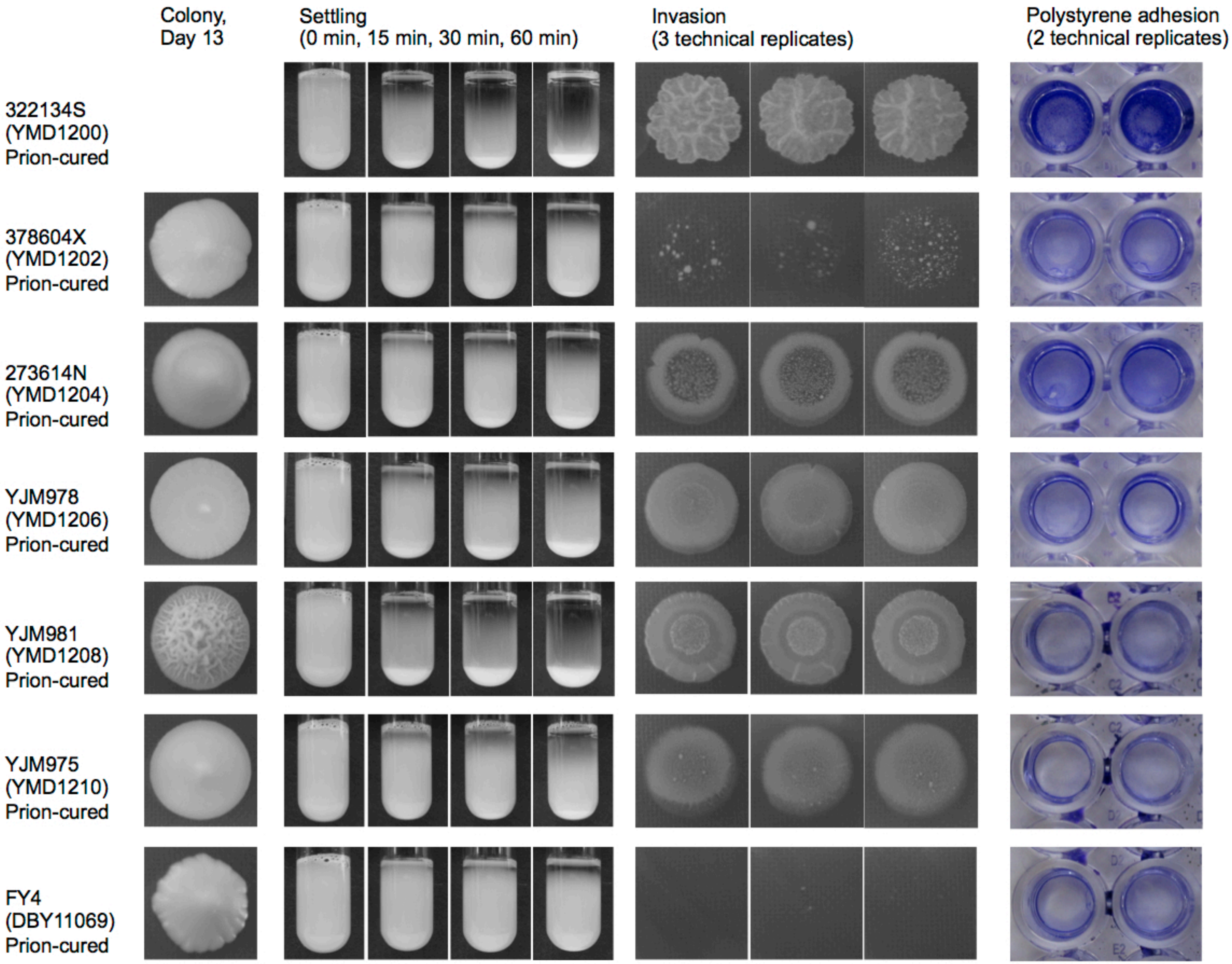

File S2: Prion-cured phenotype panel

	Colony, Day 13	Settling (0 min, 15 min, 30 min, 60 min)	Invasion (3 technical replicates)	Polystyrene adhesion (2 technical replicates)
DBVPG1853 (YMD1160) Prion-cured REPLICATE				
YPS128 (YMD1164) Prion-cured REPLICATE				
BC187 (YMD1170) Prion-cured REPLICATE				
Y12 (YMD1182) Prion-cured REPLICATE				
W303 (YMD1198) Prion-cured REPLICATE				

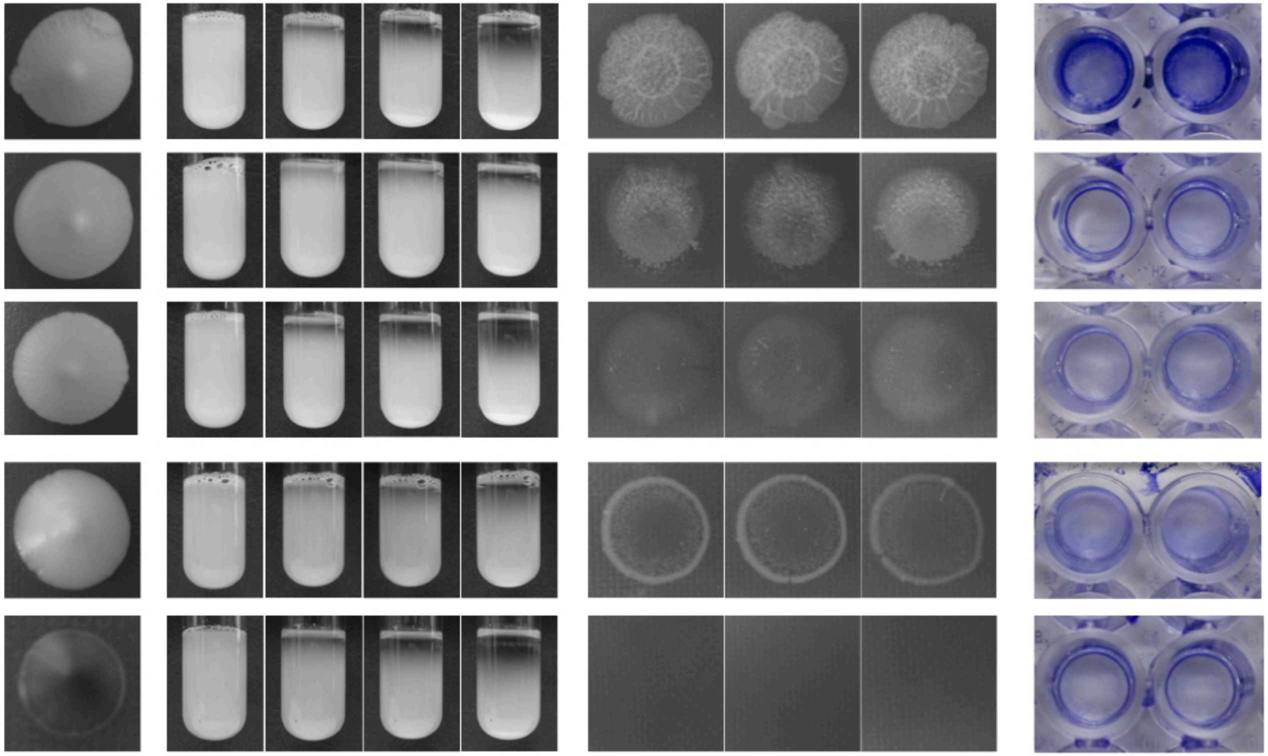

File S2 Complete prion-cured haploid phenotypic panel Full phenotypic panel for all 25 prion-cured haploid strains used in study. Strains are listed with their formal name and origin and are shown across five different phenotypes. Three technical replicates are shown for the invasion assay, photographed after 24 hours' growth following washing on day 5. Two technical replicates are shown for the polystyrene adhesion assay. Pictured biofilms are fixed and stained with a 1% w/v crystal violet solution. Cured replicates are shown as the final strains in the panel.
