# Supplementary material for: Ploidy-Regulated Variation in Biofilm-Related Phenotypes in Natural Isolates of Saccharomyces cerevisiae
Source: G3 (Bethesda). 2014 Jul 24;4(9):1773–86. doi: 10.1534/g3.114.013250 (PMC4169170; doi:10.1534/g3.114.013250)
Supplement: Supporting Information [file supp_g3.114.013250_FileS4.pdf]

File S4: Diploid phenotype panel

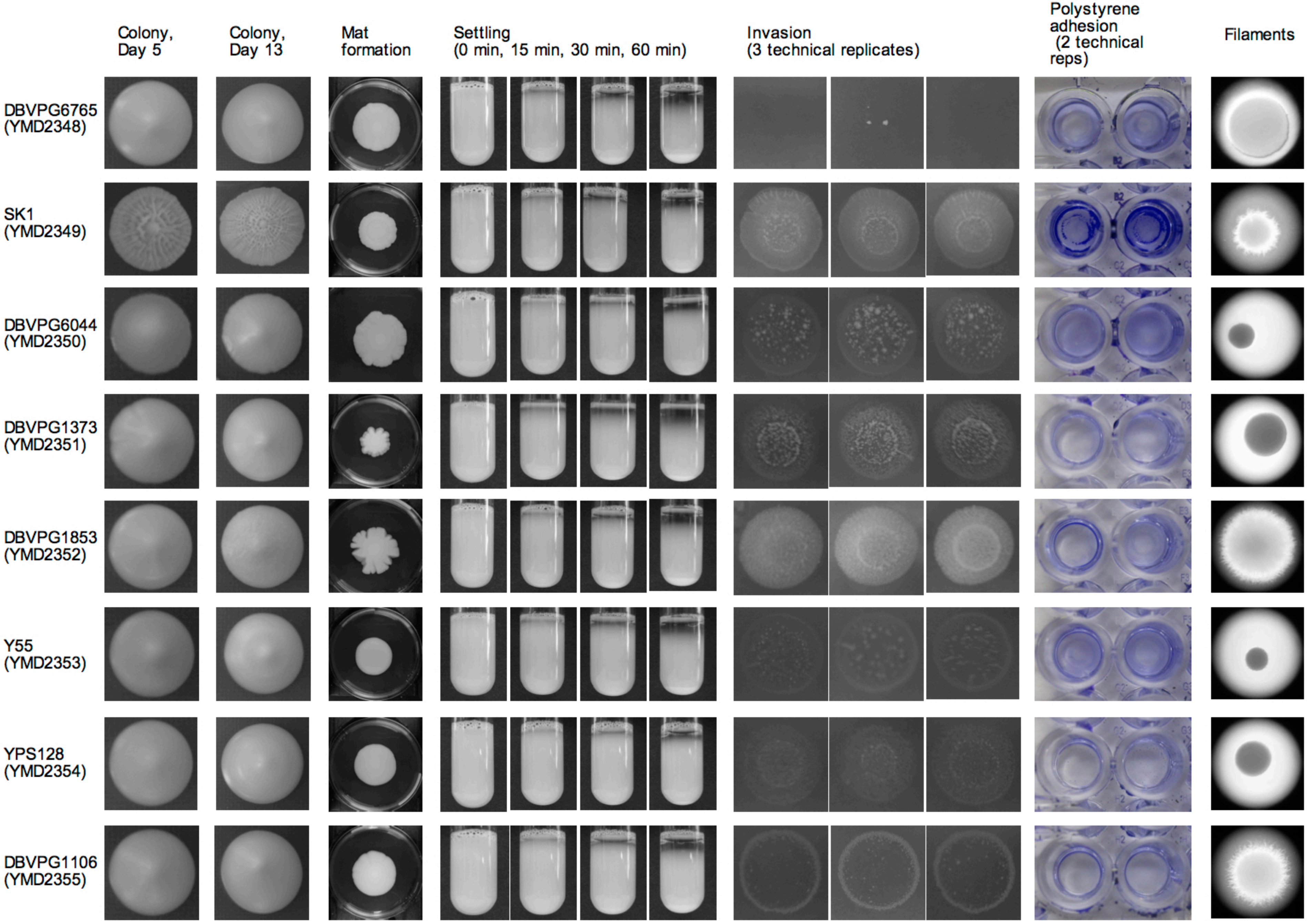

File S4: Diploid phenotype panel

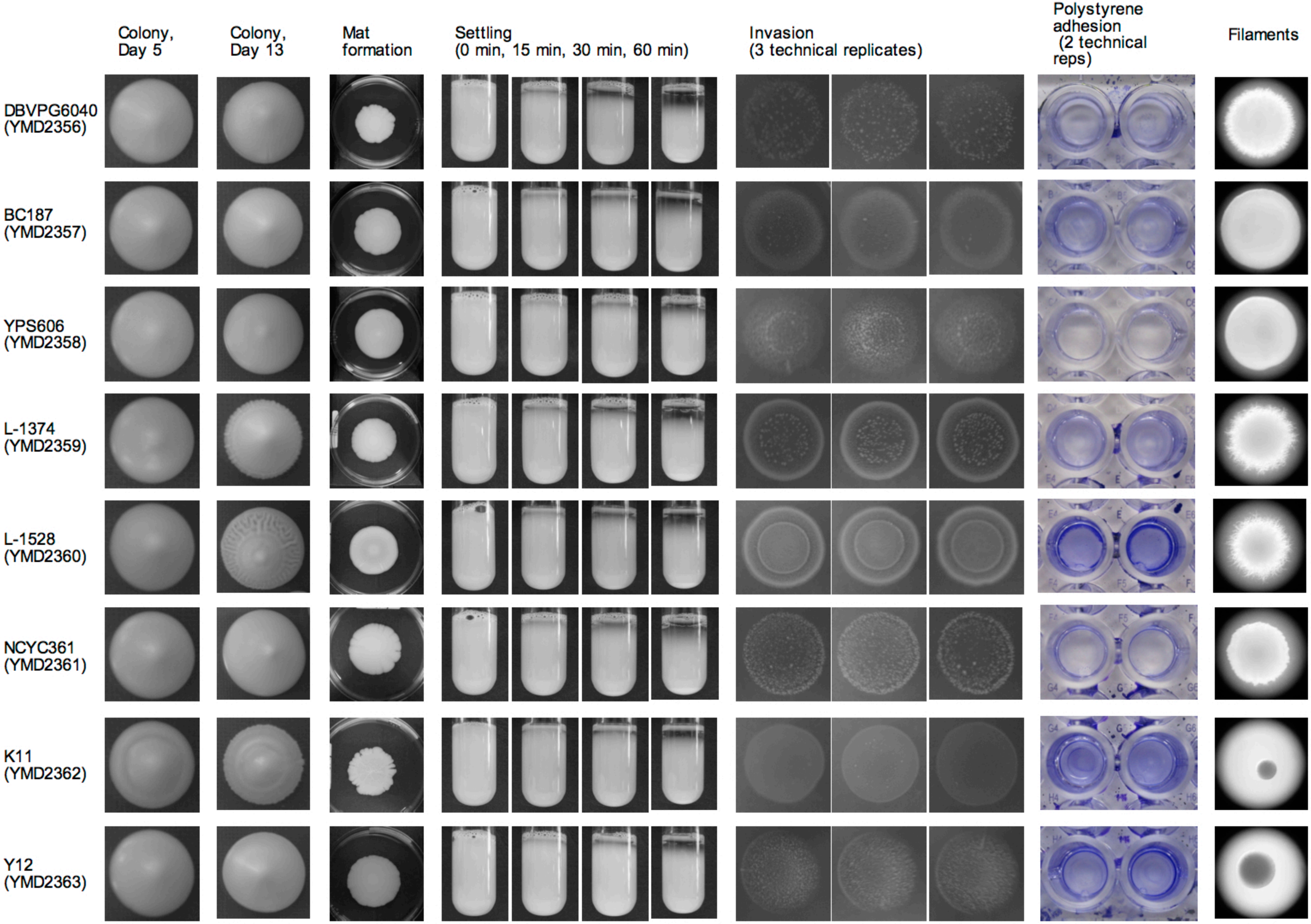

File S4: Diploid phenotype panel

	Colony, Day 5	Colony, Day 13	Mat formation	Settling (0 min, 15 min, 30 min, 60 min)	Invasion (3 technical replicates)	Polystyrene adhesion (2 technical reps)	Filaments
YS2 (YMD2364)	Diploid not tested						
YS4 (YMD2365)	Diploid not tested						
YS9 (YMD2366)	Diploid not tested						
UWOPS83-787.3 (YMD2367)	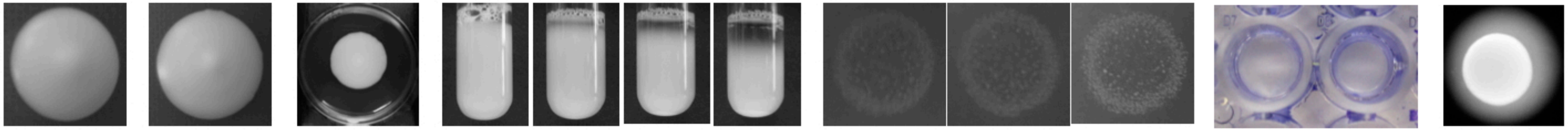						
UWOPS87-242.1 (YMD2368)	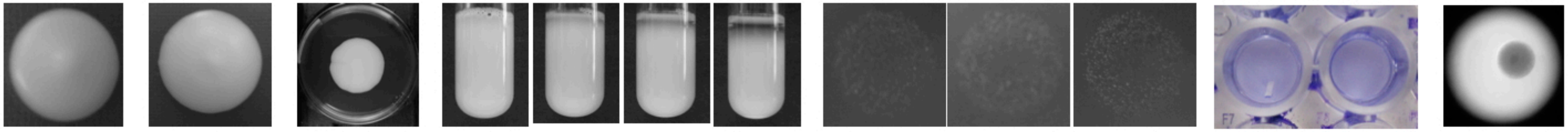						
UWOPS05-217.3 (YMD2369)	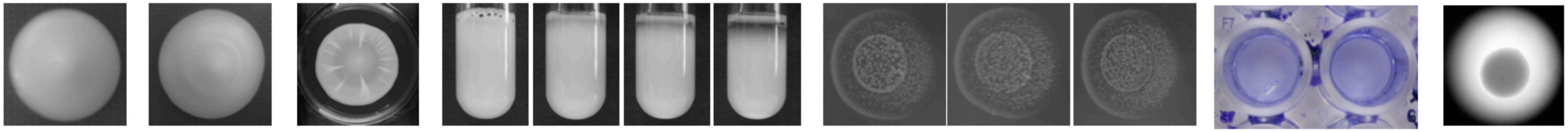						
UWOPS05-227.2 (YMD2370)	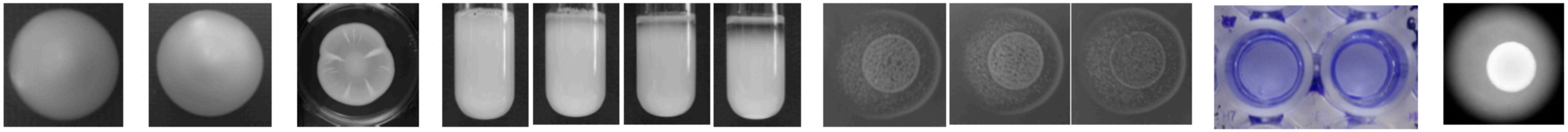						
W303 (YMD2371)	Diploid not tested						

File S4: Diploid phenotype panel

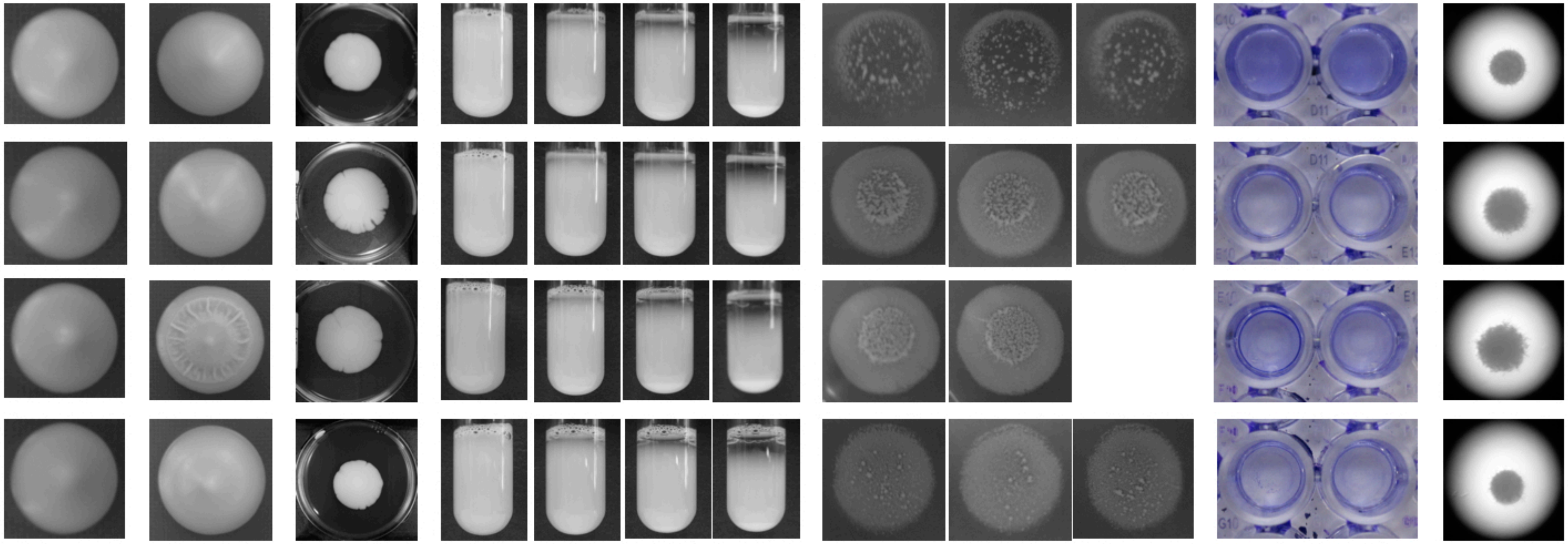

File S4 Complete diploid phenotypic panel Full phenotypic panel for all 24 diploid strains used in study. Strains are listed with their formal name and origin and are shown across six different phenotypes. Three technical replicates are shown for the invasion assay, photographed after 24 hours' growth following washing on day 5. Two technical replicates are shown for the polystyrene adhesion assay. Pictured biofilms are fixed and stained with a 1% w/v crystal violet solution.
