# Supplementary material for: Ploidy-Regulated Variation in Biofilm-Related Phenotypes in Natural Isolates of Saccharomyces cerevisiae
Source: G3 (Bethesda). 2014 Jul 24;4(9):1773–86. doi: 10.1534/g3.114.013250 (PMC4169170; doi:10.1534/g3.114.013250)
Supplement: Supporting Information [file supp_g3.114.013250_TableS1.pdf]

Table S1 ***S. cerevisiae*** **strains included in this study**

NAME	GEOGRAPHICAL ORIGIN	ECOLOGICAL ORIGIN	MATING TYPE	DIPLOID INCLUDED
DBVPG6765	Unknown	Unknown	a	yes
SK1	USA	Soil	a	yes
DBVPG6044	West Africa	Bili wine, from *Osbeckia grandiflora*	a	yes
DBVPG1373	Netherland	Soil	a	yes
DBVPG1853	Ethiopia	White Teff	a	yes
Y55	France	Grape	a	yes
YPS128	Pennsylvania, USA	Soil beneath *Q. alba*	a	yes
DBVPG1106	Australia	Grapes	a	yes
DBVPG6040	Netherland	Fermenting fruit juice	a	yes
BC187	Napa Valley, USA	Barrel fermentation	a	yes
YPS606	Pennsylvania, USA	Bark of *Q. rubra*	a	yes
L-1374	Chile	Fermentation from must Pais	a	yes
L-1528	Chile	Fermentation from must Cabernet	a	yes
NCYC361	Ireland	Beer spoilage strain from wort	a	yes
K11	Japan	Shochu sake strain	a	yes
Y12	Africa - Ivory Coast	Palm wine strain	a	yes
YS2	Australia	Baker strain	a	no
YS4	Netherlands	Baker strain	a	no
YS9	Singapore	Baker strain	a	no
UWOPS83-787.3	Bahamas	Fruit, *Opuntia stricta*	a	yes
UWOPS87-2421	Hawaii	Cladode, *Opuntia megacantha*	a	yes
UWOPS05-217.3	Malaysia	Nectar, Bertram palm	a	yes
UWOPS05-227.2	Malaysia	*Trigona* spp (Stingless bee) collected near Bertram palm flower	a	yes
W303	Unknown	N/A	a	no
322134S	RVI, Newcastle, UK	Clinical isolate (throat sputum)	a	no
378604X	RVI, Newcastle, UK	Clinical isolate (sputum)	a	no
273614N	RVI, Newcastle, UK	Clinical isolate (fecal)	a	yes

YJM978	Bergamo, Italy	Clinical isolate (vaginitis)	a	yes
YJM981	Bergamo, Italy	Clinical isolate (vaginitis)	a	yes
YJM975	Bergamo, Italy	Clinical isolate (vaginitis)	a	yes
FY4	Laboratory strain	Laboratory strain	a	no
