# Supplementary material for: Ploidy-Regulated Variation in Biofilm-Related Phenotypes in Natural Isolates of Saccharomyces cerevisiae
Source: G3 (Bethesda). 2014 Jul 24;4(9):1773–86. doi: 10.1534/g3.114.013250 (PMC4169170; doi:10.1534/g3.114.013250)
Supplement: Supporting Information [file supp_g3.114.013250_TableS2.pdf]

Table S2 Qualitative scoring metrics

ASSAY	SCORE	CRITERIA
Complex colony morphology	0	simple colonies
	1	non-smooth colony surface
	2	signs of complex morphology
	3	moderate complex morphology (does not cover entire colony)
	4	strong complex morphology (covers entire colony)
	5	very strong complex morphology
Complex mat formation	0	simple mat
	1	very light sectoring and/or light ruffle on edge
	2	clear ruffle on edge or petals, can include sectoring
	3	obvious complexity at edges
	4	obvious complexity at edges and in center
	5	strong complexity across entirety of mat
Diploid filamentous growth	0	no filaments
	1	short filaments sparsely distributed around perimeter
	2	long filaments sparsely distributed around perimeter
	3	long filaments distributed around majority of perimeter
	4	moderate length filaments densely distributed around entire perimeter
	5	long filaments densely distributed around entire perimeter
