# Supplementary material for: Ploidy-Regulated Variation in Biofilm-Related Phenotypes in Natural Isolates of Saccharomyces cerevisiae
Source: G3 (Bethesda). 2014 Jul 24;4(9):1773–86. doi: 10.1534/g3.114.013250 (PMC4169170; doi:10.1534/g3.114.013250)
Supplement: Supporting Information [file supp_g3.114.013250_TableS3.pdf]

Table S3 Qualitative score assignments

STRAIN	HAPLOID BIOREP1 COLONY	HAPLOID BIOREP2 COLONY	HAPLOID BIOREP1 MAT	DIPLOID COLONY	DIPLOID MAT
DBVPG6765	0	0	0	0	1
SK1	4	4	4	4	4
DBVPG6044	2	2	2	1	4
DBVPG1373	0	0	0	0	3
DBVPG1853	1	2	0	1	3
Y55	1	0	2	2	0
YPS128	3	3	4	2	0
DBVPG1106	0	0	0	0	1
DBVPG6040	4	3	4	1	2
BC187	2	2	5	0	1
YPS606	3	3	5	0	0
L-1374	3	3	4	2	2
L-1528	4	5	5	3	3
NCYC361	0	0	0	0	3
K11	3	3	5	3	4
Y12	0	0	0	0	1
YS2	0	0	1		
YS4	1	1	3		
YS9	0	0	2		
UWOPS83-787.3	1	1	0	0	0
UWOPS87-2421	1	1	0	1	0
UWOPS05-217.3	5	5	5	2	4
UWOPS05-227.2	5	5	5	1	4
W303	0	0	2		
322134S	5		5		
378604X	1	1	2		
273614N	1	1	0	0	1
YJM978	3	2	5	0	3
YJM981	5	5	5	3	2
YJM975	0	0	1	2	2
FY4	2	2	3		

Strains are assigned qualitative scores based on the metric described in Table S2.
