# Supplementary material for: Ploidy-Regulated Variation in Biofilm-Related Phenotypes in Natural Isolates of Saccharomyces cerevisiae
Source: G3 (Bethesda). 2014 Jul 24;4(9):1773–86. doi: 10.1534/g3.114.013250 (PMC4169170; doi:10.1534/g3.114.013250)
Supplement: Supporting Information [file supp_g3.114.013250_TableS4.pdf]

Table S4 Quantitative changes in prion-cured strains vs. haploid strains

	FLOCCULATION[a]	INVASION[b]	ADHESION[c]
Significant decrease by FDR correction*	5	1	0
No change by FDR correction	20	20	23
Significant increase by FDR correction	1	2	2

The numbers of prion-cured strains that showed differences when compared to the original haploid strains across three quantitative metrics are shown. Quantitative values were compared using a two-tailed t-test assuming unequal variance across all technical replicates for each strain. *P*-values were evaluated for significance using the *q*-values package from Storey with the Benjamini-Hochberg method (Storey 2002).

* FDR = 0.05
[a] 26 strains
[b] 23 strains
[c] 25 strains
